# Supplementary material for: Rapidly sequence-controlled electrosynthesis of organometallic polymers
Source: Nat Commun. 2020 May 21;11:2530. doi: 10.1038/s41467-020-16255-z (PMC7242481; doi:10.1038/s41467-020-16255-z)
Supplement: Supplementary file 1 — Supplementary Information [file 41467_2020_16255_MOESM1_ESM.pdf]

Supplementary Information for

Rapidly Sequnce-Controlled Electrosynthesis of Organometallic Polymers

J. Zhang et al.

Supplementary Figures

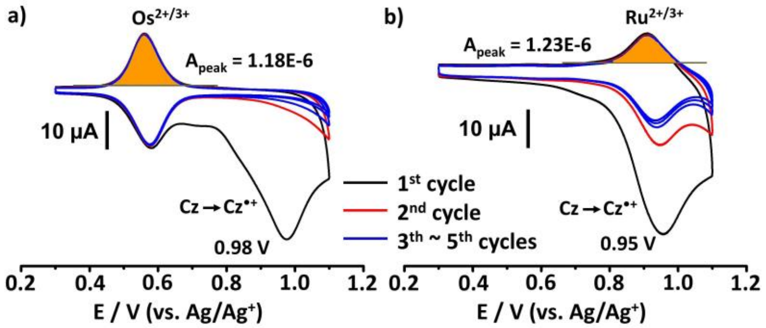

Supplementary Fig. 1 Successive CVs for electrochemical chacterazation. a Self-assembled Os^{II}PX. **b** Self-assembled Ru^{II}PX. Working electrode is ITO coated glass. Scan rate is 100 mV/s in 0.10 M Bu_4NClO_4/MeCN monomer free electrolyte. Strong irreversible oxidation peaks of carbazolyl appear at 0.95 ~ 0.98 V, and the redox peaks of 3,3′-bicarbazolyls[1,2] at 0.50 ~ 0.60 V cannot be observed, implying that there are no self-coupling reactions of carbazolyls within the self-assembled monolayer. Usually, the redox peaks of 3,3′-bicarbazolyls at 0.50 ~ 0.60 V can be clearly observed (Figures in Supplementary Ref. 1 and Ref. 2).

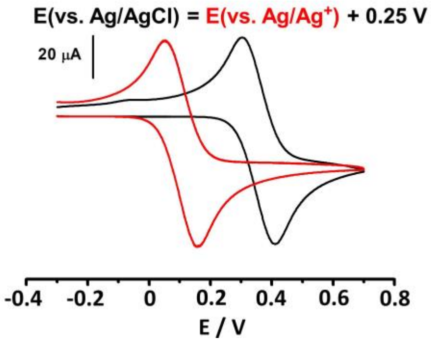

Supplementary Fig. 2 Potential calibrating. Working electrode is glassy carbon in 0.10 M Bu_4NClO_4/MeCN solution according to different reference electrodes at scan rate of 100 mV/s. Ag/Ag^+ was used during iterative synthesis and CV measurements. Ag/AgCl was used for in situ optoelectric measurements. Here red and black lines are for Ag/Ag^+ and Ag/AgCl, respectively.

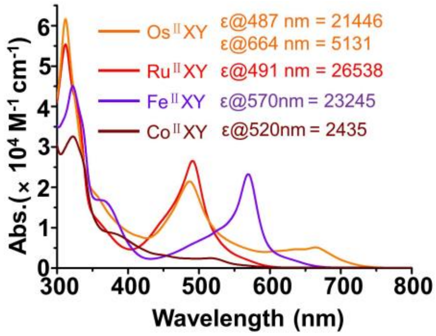

Supplementary Fig. 3 Molar extinction coefficient. Absorption spectra of $M^{II}XY$ (M = Os^{II}, Fe^{II} and Ru^{II}). Concentration is 4.0 × 10^{-2} mM MeCN solution. The intense high energy bands of all complexes in UV region between 300 nm and 400 nm (shoulder) are dominated by π−π* and internal transitions of the coordinated ligands.[3] Moderate absorption bands in the range of 490−600 nm are attributed to metal-to-ligand [M^{II}(dπ) to tpy(π*)] charge transfer (MLCT) transition.[3] It is of interest to note that absorption band at 680 nm seems to be due to spin-forbidden MLCT transition from $^1[Os^{II}(d\pi)^6]$ to $^3[Os^{II}(d\pi)^5tpy(\pi^*)^1]$.[4]

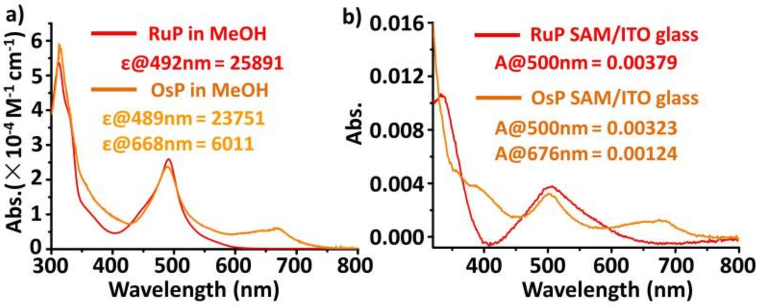

Supplementary Fig. 4 Molar extinction coefficient. **a** Absorption spectra of $M^{II}PX$ (M = Os and Ru) in 4.0 × 10^{-2} mM MeOH solution. **b** Absorption spectra of self-assembled $M^{II}PX$ (M = Os and Ru). Surface coverage (Γ) was calculated by using the format: $\Gamma = A(\lambda_{peak})/\varepsilon(\lambda_{peak})/1000$, thus obtained Γ(self-assembled $Ru^{II}PX$) = 1.46 × 10^{-10} mol cm^{-2}, Γ(self-assembled $Os^{II}PX$) = 1.36 × 10^{-10} mol cm^{-2}.

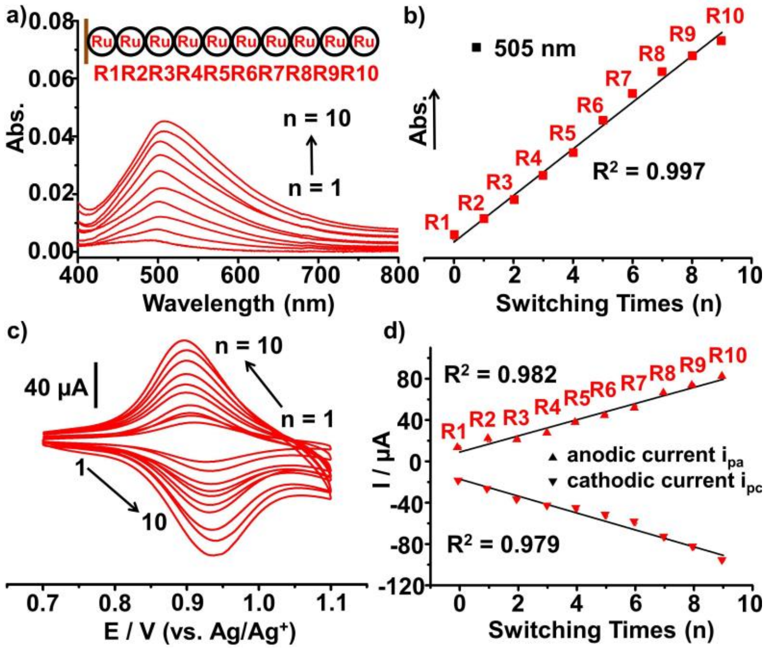

Supplementary Fig. 5 Electrosynthesis of self-assembled Ru^{II} organometallic polymers. a, b Illustrations of UV-vis absorbance and intensities as function of switching times during iterative synthesis. **c, d** Illustrations of CV and its intensities of redox peaks as function of switching times during iterative synthesis.

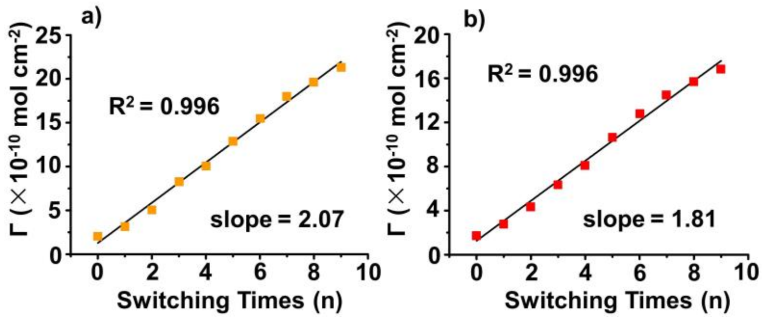

Supplementary Fig. 6 Surface coverage. a Surface coverage of Os^{II} organometallic polymers. **b** Surface coverage of Ru^{II} organometallic polymers. These values were calculated by $\Gamma = A(\lambda_{peak})/\varepsilon(\lambda_{peak})/1000$ as function of switching times of oxidative and reductive reactions.

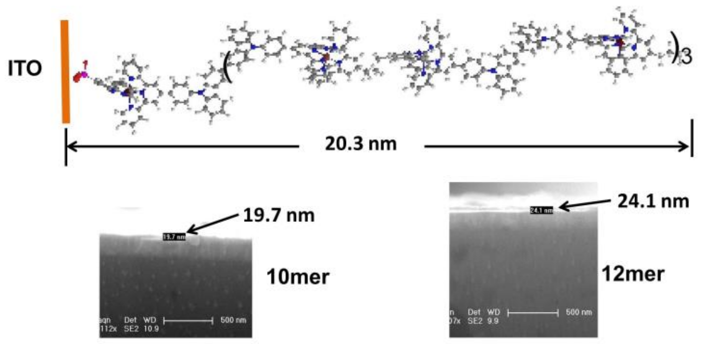

Supplementary Fig. 7 Calculations of molecular lengths. Theoretical length of Ru^{II} organometallic polymer with 10 units and the thicknesses of assembled films in SEM images. There is 4.4 nm between thicknesses of 10-mer and 12-mer, in good agreements with iterative synthesis in single monomer precise.

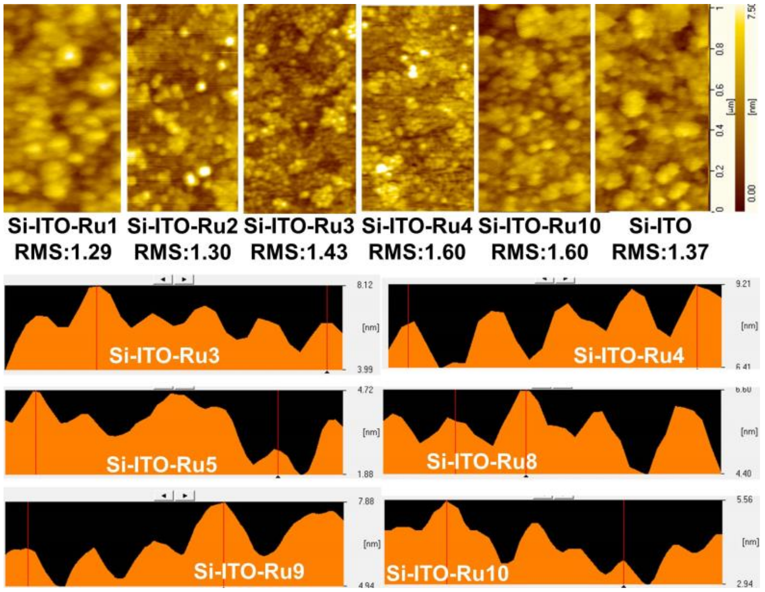

Supplementary Fig. 8 AFM observations. AFM images of self-assembled Ru^{II} organometallic polymers with different lengths, and height differences of organometallic polymers as solid film in AFM images. All images have similar roughness of morphology, and the height differences in all images are less than 2.0 nm, which is similar to the length of single monomer, in good agreement with iterative synthesis in single monomer precise.

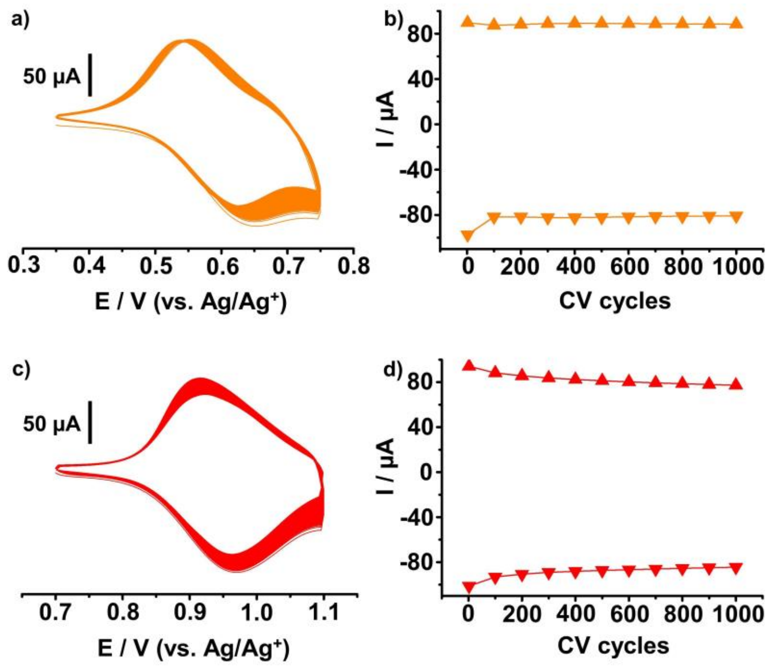

Supplementary Fig. 9 Electrochemical stability. a, b CVs from 1st to 1000th and the current intensities as function of CV cycles for self-assembled Os^{II} organometallic polymers. **c, d** CVs from 1st to 1000th and the current intensities as function of CV cycles for self-assembled Ru^{II} organometallic polymers. The current intensities of redox peaks for both organometallic polymers do not show significant change from 2nd to 1000th cycles in open air, indicating these organometallic polymers have good electrochemical stability.

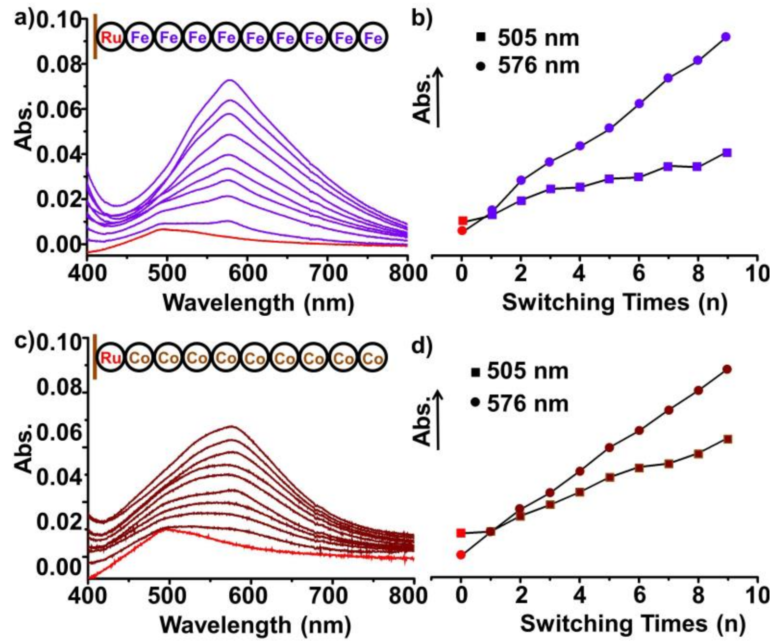

Supplementary Fig. 10 Electrosynthesis of organometallic polymers on assembled Ru^{II}. a, **b** Electrosynthesis of Fe^{II} organometallic polymers monitored by UV-vis spectra with relationships of their intensities as function of switching times of oxidative and reductive reactions. **c**, **d** Electrosynthesis of Co^{II} organometallic polymers monitored by UV-vis spectra with relationships of their intensities as function of switching times of oxidative and reductive reactions.

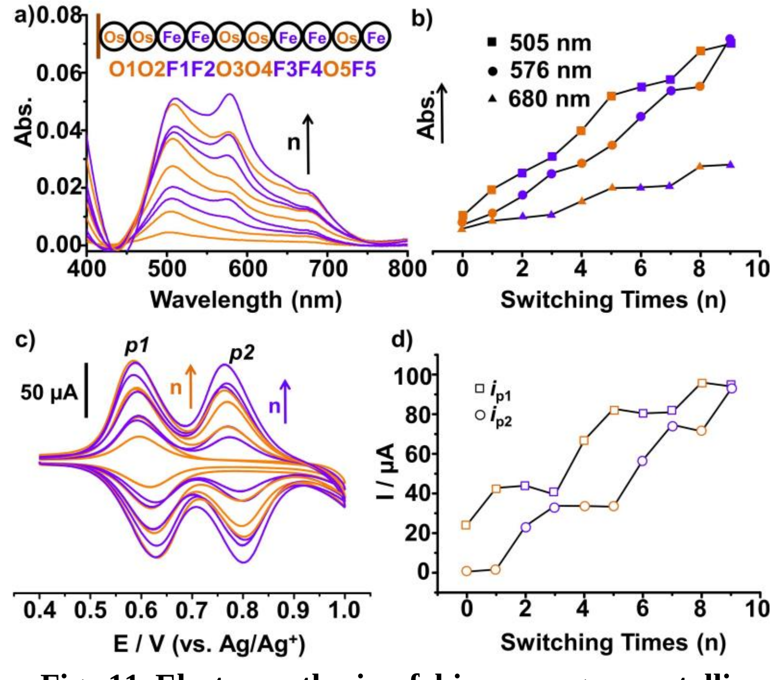

Supplementary Fig. 11 Electrosynthesis of binary organometallic polymers. a, b Illustrations of UV-vis absorbance and intensities as function of switching times during

iterative synthesis. **c**, **d** Illustrations of current intensities of oxidative peaks as function of switching times during iterative synthesis.

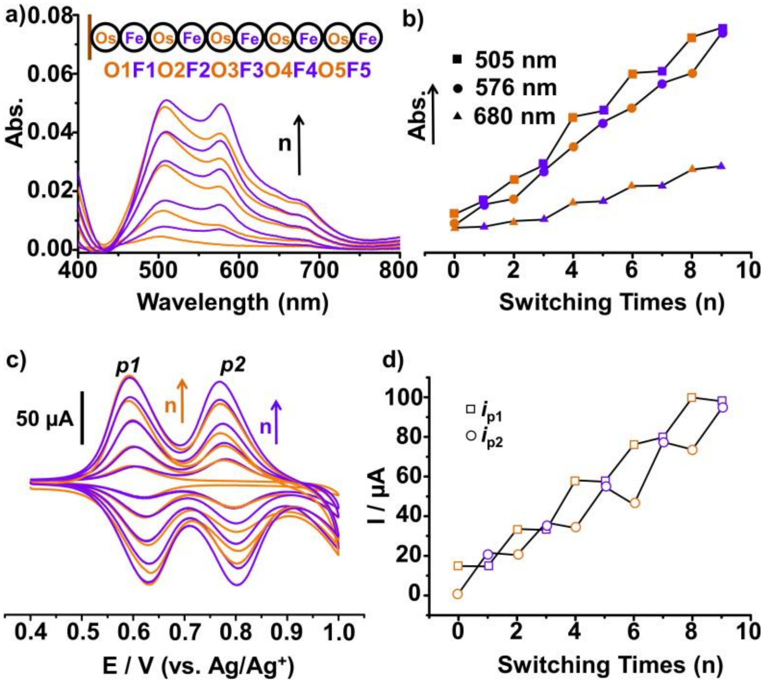

Supplementary Fig. 12 Electrosynthesis of binary organometallic polymers. a, b Illustrations of UV-vis absorbance and intensities as function of switching times during iterative synthesis. **c**, **d** Illustrations of current intensities of oxidative peaks as function of switching times during iterative synthesis.

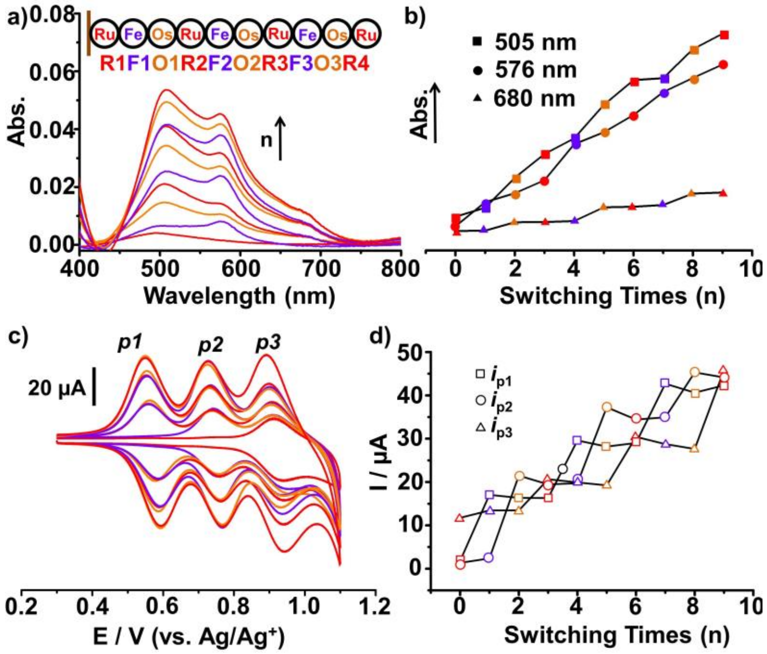

Supplementary Fig. 13 Electrosynthesis of ternary organometallic polymers. a, b Illustrations of UV-vis absorbance and intensities as function of switching times during iterative synthesis. **c**, **d** Illustrations of current intensities of oxidative peaks as function of switching times during iterative synthesis.

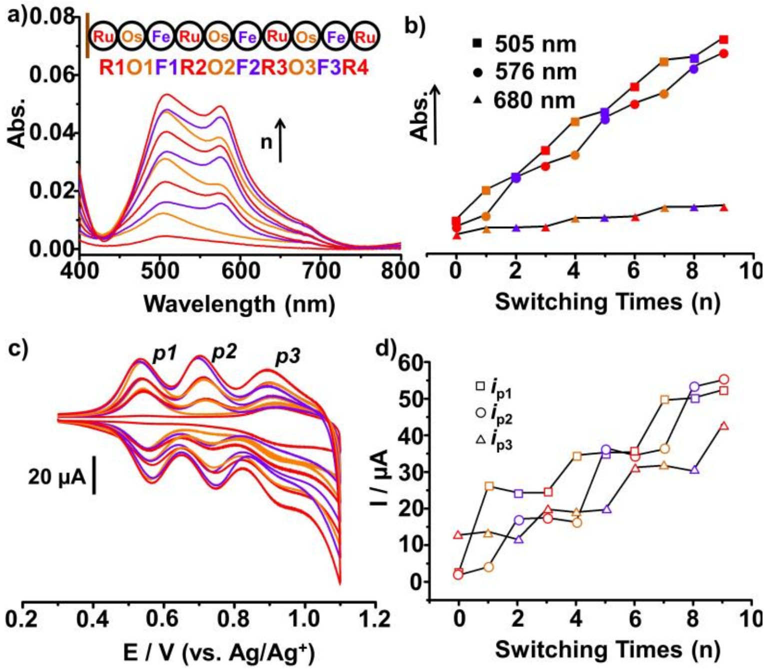

Supplementary Fig. 14 Electrosynthesis of ternary organometallic polymers. a, b Illustrations of UV-vis absorbance and intensities as function of switching times during

iterative synthesis. **c**, **d** Illustrations of current intensities of oxidative peaks as function of switching times during iterative synthesis.

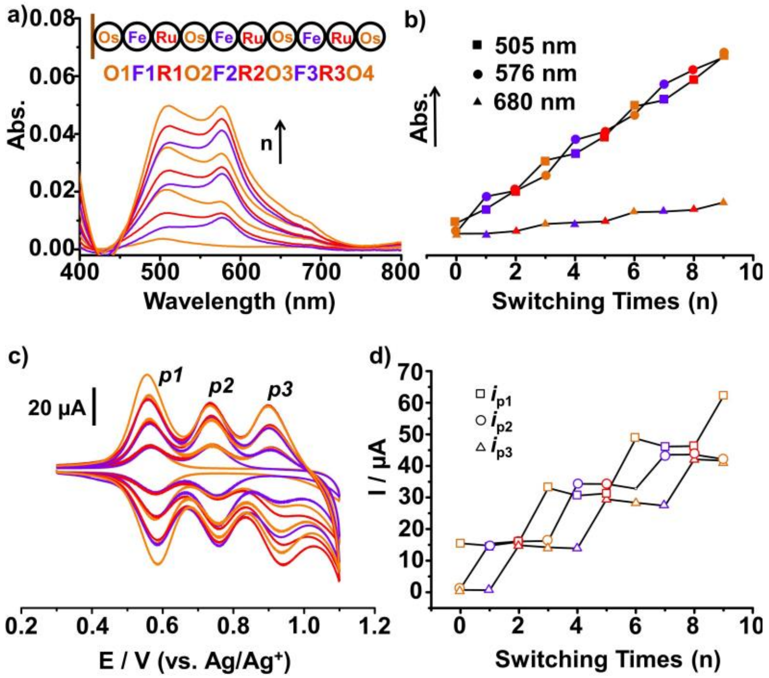

Supplementary Fig. 15 Electrosynthesis of ternary organometallic polymers. a, b Illustrations of UV-vis absorbance and intensities as function of switching times during iterative synthesis. **c**, **d** Illustrations of current intensities of oxidative peaks as function of switching times during iterative synthesis.

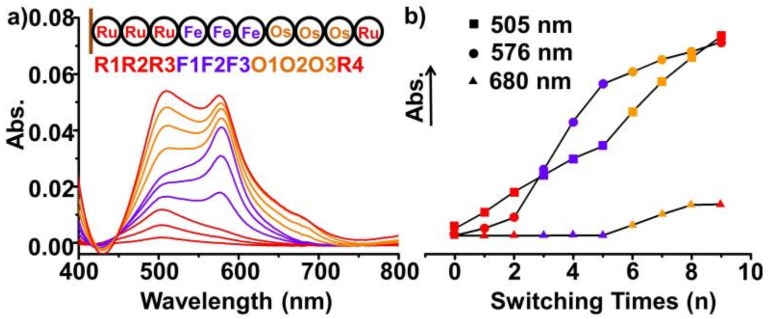

Supplementary Fig. 16 Electrosynthesis of ternary organometallic polymers. a, b Illustrations of UV-vis absorbance and intensities as function of switching times during iterative synthesis.

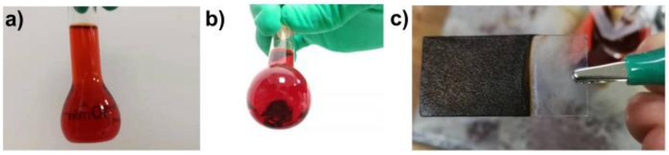

Supplementary Fig. 17 Photos for observations. a, b Photos of Ru^II^XY solutions before and after iterative synthesis. **c** Photo of bare ITO electrode without pre-assmebled molecule after electrolysis of Ru^II^XY at 1.0 V for 1h. The precipitation can be found after interative synthesis, indicating the oligomers become insoluble, and also implying that the single organometallic polymer with 10 units will be hard to be soluble. After electrolysis of Ru^II^XY at 1.0 V for 1h, the dark red film was found on ITO surface (c) and directly dissolved for measurements of NMR and Mass spectra as shown in Supplementary Fig. 18,19, demonstrating that this film was mainly composed of dimer with bad solubility. We have tried to get the isolated products from excess supporting electrolytes mostly via silica gel chromatography. This experiment failed because the dimer with counterion is eluted out along with $Bu_4NH_4ClO_4$.

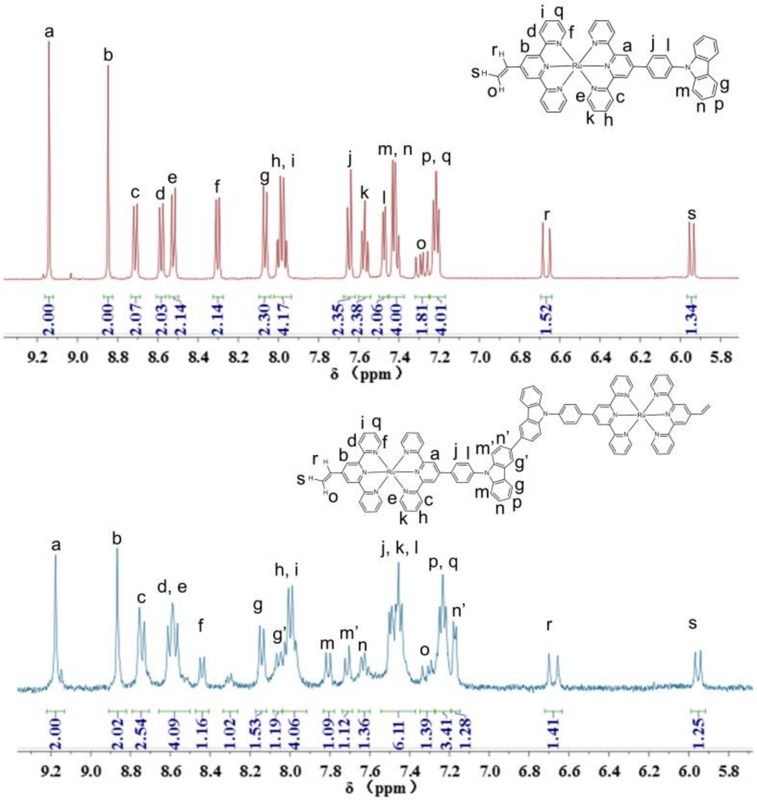

Supplementary Fig. 18 Structural characterization. ^{1}H NMR spectra of Ru^II^XY and its dimer in CD_3CN obtained by electrolysis in Supplementary Fig. 17c without any purification. Regarding the impurity feature, the unreactive monomer could possibly and

physically co-deposite into film during continuously electrolysis of 1h. We did not find the clear evidence that the carbazole coupling at 1.0 V is forming regioisomers for these monomers or other monomers, which we were and are studying on. Herein, this possibility should be limited in iterative electrosynthesis because of steric hindrance within organometallic polymers. Additionally, it is well-known that the purity will decrease in case of large scale synthesis. Compared to synthesis on 1.0 cm^2 substrate (10^{-10} mol and 10^{-7} g for each step in Supplementary Fig. 4), the experiment in Supplementary Fig. 18 (c.a. 2 mg) was enlarged in over 10000 times. Iterative synthesis in manuscript took place at a distance of 20 nm from electrode surface, while this electrodeposition fabricated the film with probable thickness of >10 μm. Therefore, the coupling ratio in Supplementary Fig. 18 and the coupling ratio of each step for iterative synthesis of organometallic polymers could not be simply compared. The relationship should be an clear curve even if there was significant decrease in total conversion (c.a. 10% after 10th coupling). The relationship between units and steps in Fig. 2 and Supplementary Fig. 6 shows excellent linear, demonstrating that there is no significant change in conversion yield.

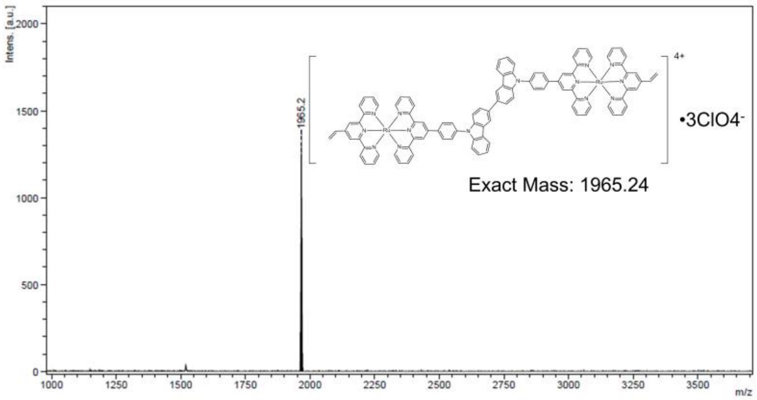

Supplementary Fig. 19 Structural characterization. MALDI-TOF mass spectrum of Ru^{II}XY dimer directly obtained by electrolysis in Supplementary Fig. 17c without any purification.

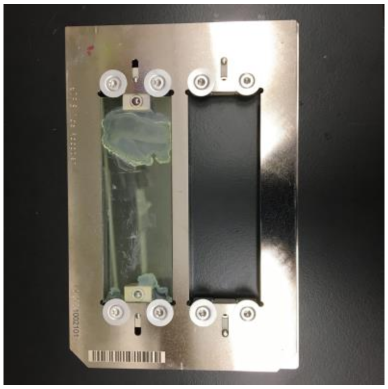

Supplementary Fig. 20 Photo of sample for Mass spectra. Organometallic polymers of Os and Ru at ITO coated glass (2.5 cm × 7.5 cm) for measurements of MALDI-TOF mass spectra. In the experiment, DCTB (20 mg/mL) was used as matrix and spotted on the polymers coated ITO (2.5 cm × 2.0 cm). The calibration was carried out using PMMA (polymethyl methacrylate). Both reflection and linear modes were used to obtain signals. Fe core was not incorporated into organometallic polymers because its weak coordination[5] is unstable compared to Os and Ru cores. Herein, we have prepared 3 types of Ru and Os organometallic polymers (1111122222, 1122112212, 1212121212, 1 = Ru, 2 = Os). Ru coordination is more stable than Os coordination in Supplementary Fig. 21-23. Mass does not reach the theoretical value probably due to weak coordination, which could lead to more small fragments and also result in deviation in Ru and Os alternative organometallic polymers, while there is extremely tiny polymers on ITO substrate. Therefore, the alternative Ru and Os organometallic polymers are not well-recognized in main peaks of their mass spectra (Supplementary Fig. 24-28).

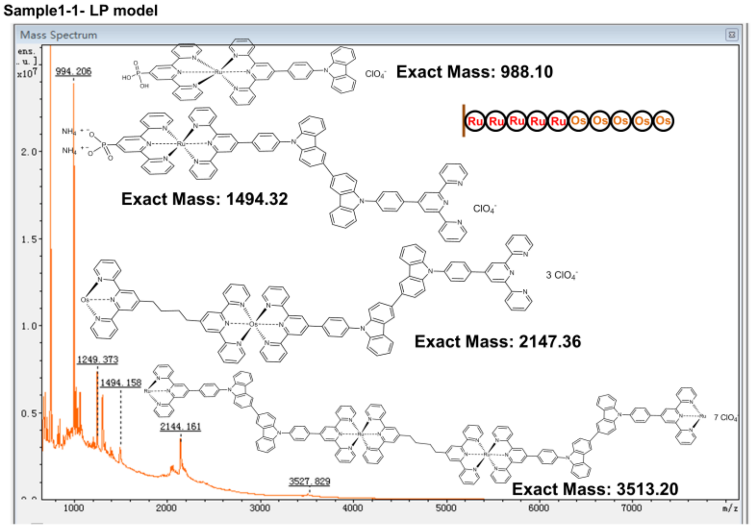

Supplementary Fig. 21 Structural characterization. MALDI-TOF mass spectrum of Os^{II} and Ru^{II} organometallic polymers in linear mode.

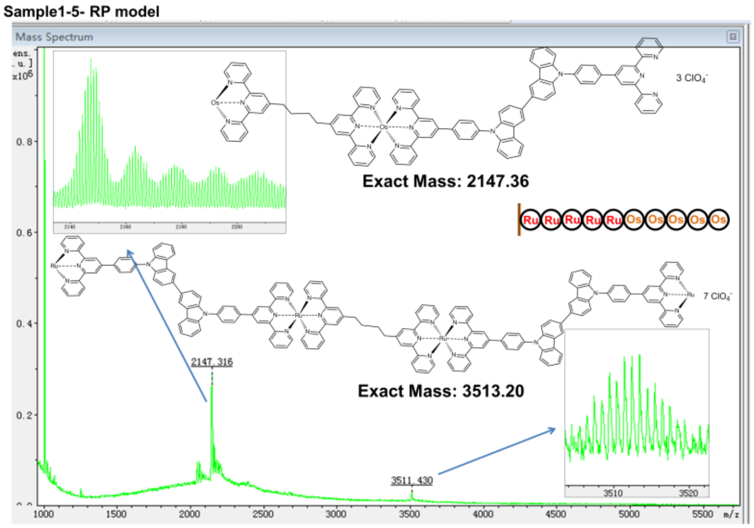

Supplementary Fig. 22 Structural characterization. MALDI-TOF mass spectrum of Os^{II} and Ru^{II} organometallic polymers in reflection mode. Molecular fragments of organometallic polymers with theoretical mass of 2147.36 and 3513.20 are found in mass spectrum, and they can be clearly identified. These two fragments contain two Os cores and four Ru cores, respectively, indicating a diblock pattern of original copolymers. The broken coordination bonds between metal cores and ligands are deemed an obstacle to obtain the direct evidence of intact coordination polymers by mass spectrometry. Two fragments contain both structures of carbazolyl and vinyl dimerization, clarifying the elementary reactions of electrochemical iterative synthesis.

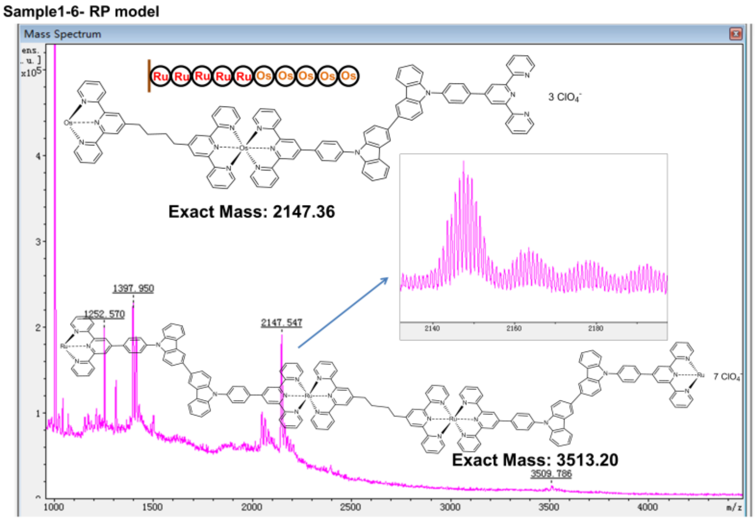

Supplementary Fig. 23 Structural characterization. MALDI-TOF mass spectrum of Os^{II} and Ru^{II} organometallic polymers in reflection mode.

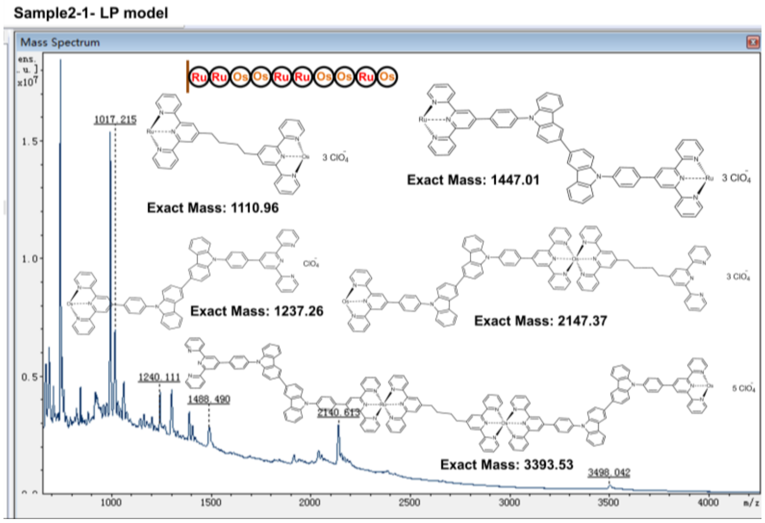

Supplementary Fig. 24 Structural characterization. MALDI-TOF mass spectrum of Os^{II} and Ru^{II} organometallic polymers in linear mode.

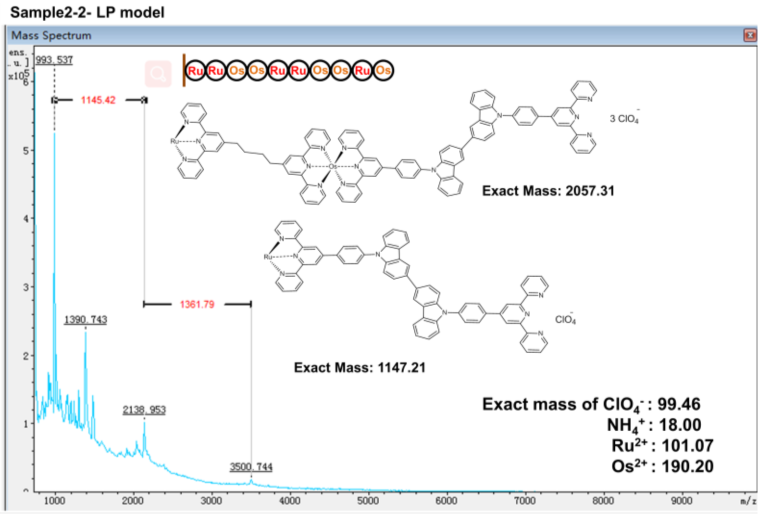

Supplementary Fig. 25 Structural characterization. MALDI-TOF mass spectrum of Os^{II} and Ru^{II} organometallic polymers in linear mode.

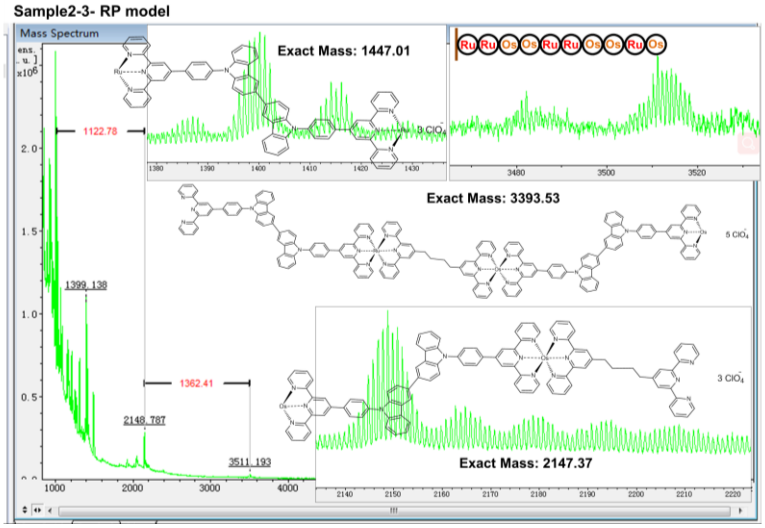

Supplementary Fig. 26 Structural characterization. MALDI-TOF mass spectrum of Os^{II} and Ru^{II} organometallic polymers in reflection mode.

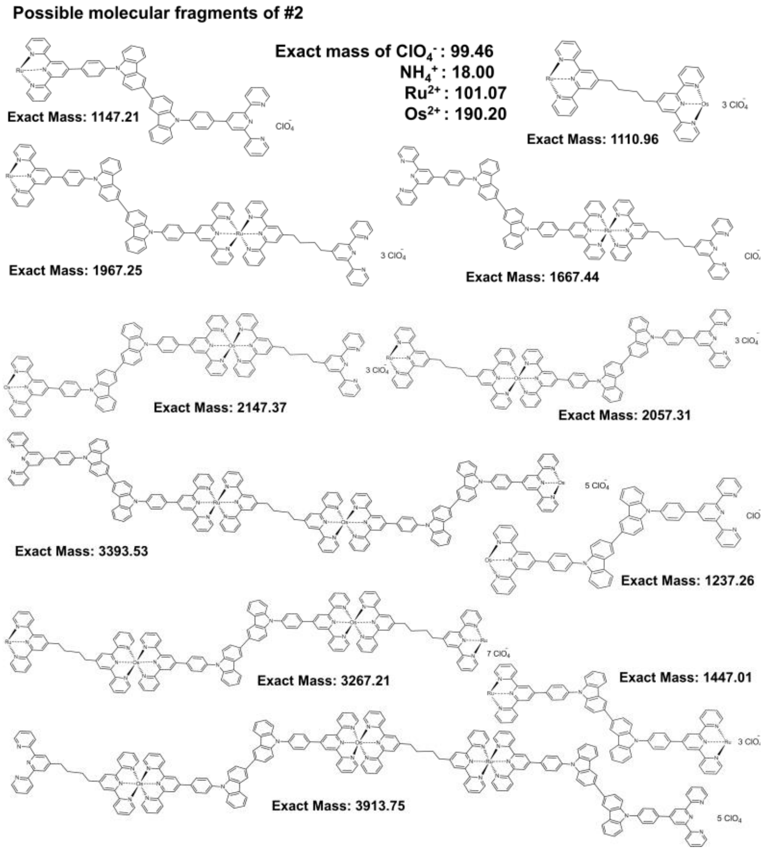

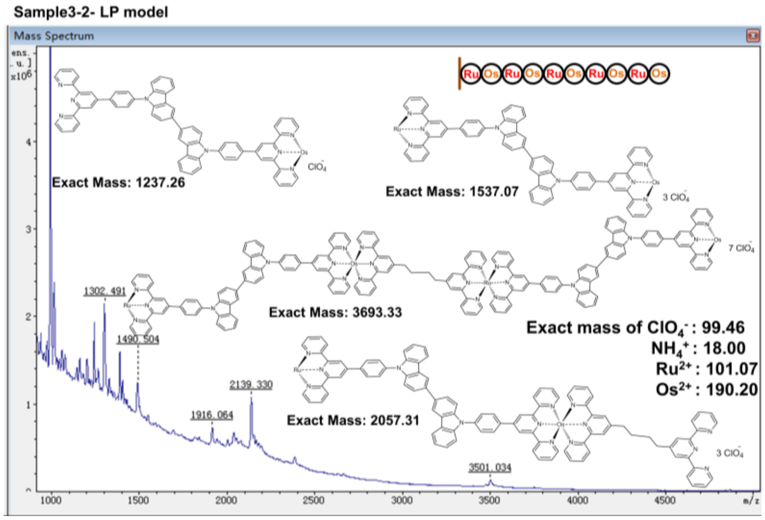

Supplementary Fig. 27 Structural characterization. MALDI-TOF mass spectrum of Os^{II} and Ru^{II} organometallic polymers in linear mode.

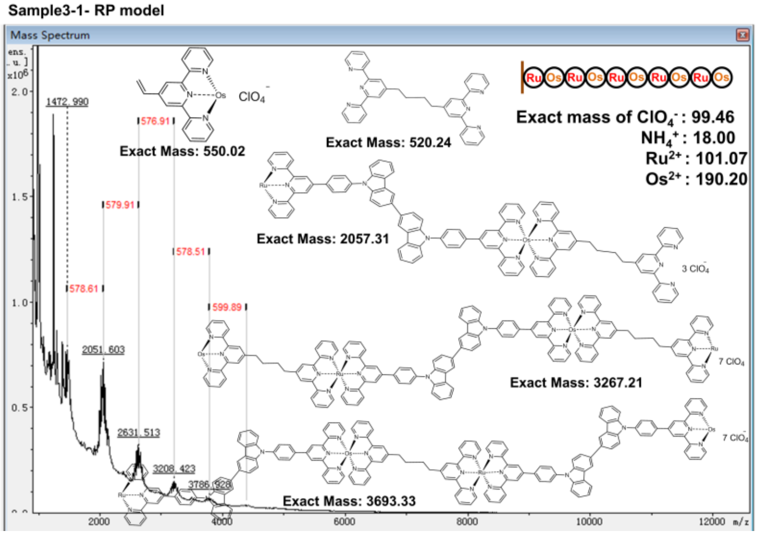

Supplementary Fig. 28 Structural characterization. MALDI-TOF mass spectrum of Os^{II} and Ru^{II} organometallic polymers in reflection mode.

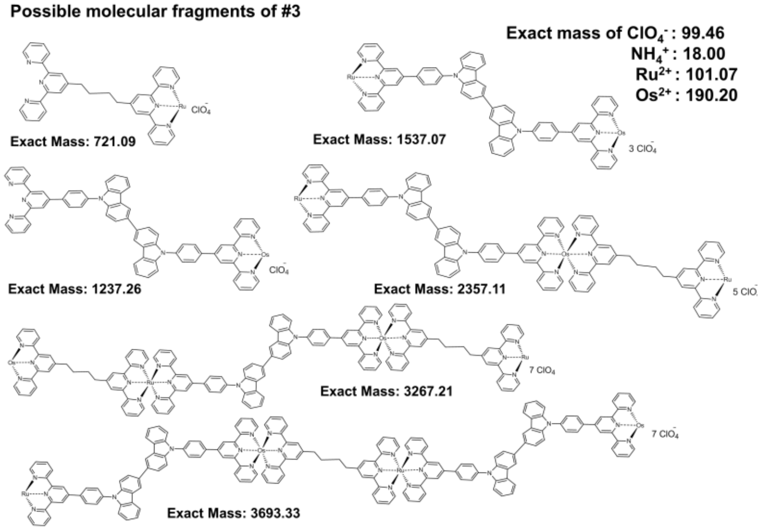

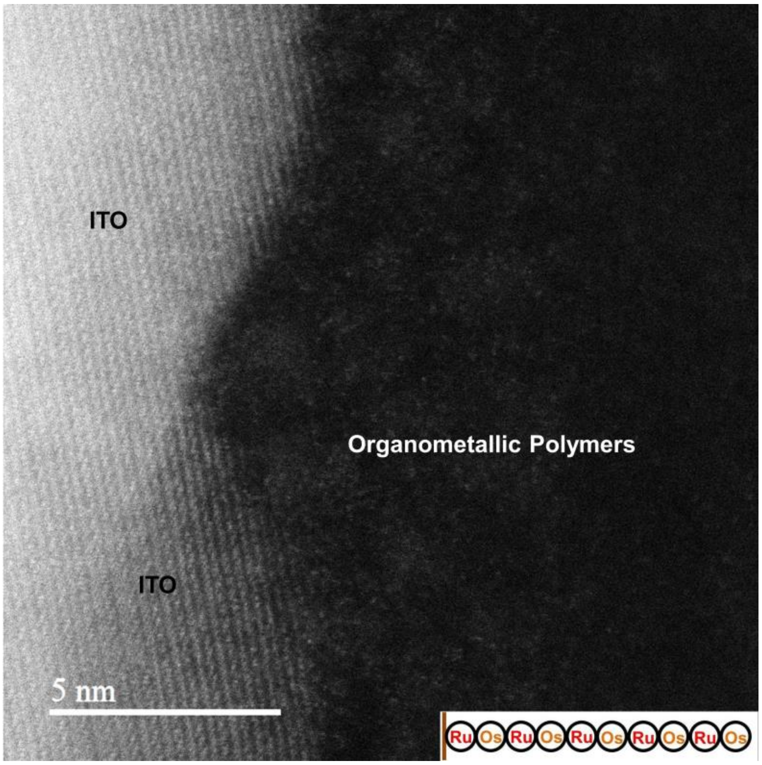

Supplementary Fig. 29 Structural characterization. STEM image of Os^{II} and Ru^{II} alternative organometallic polymers.

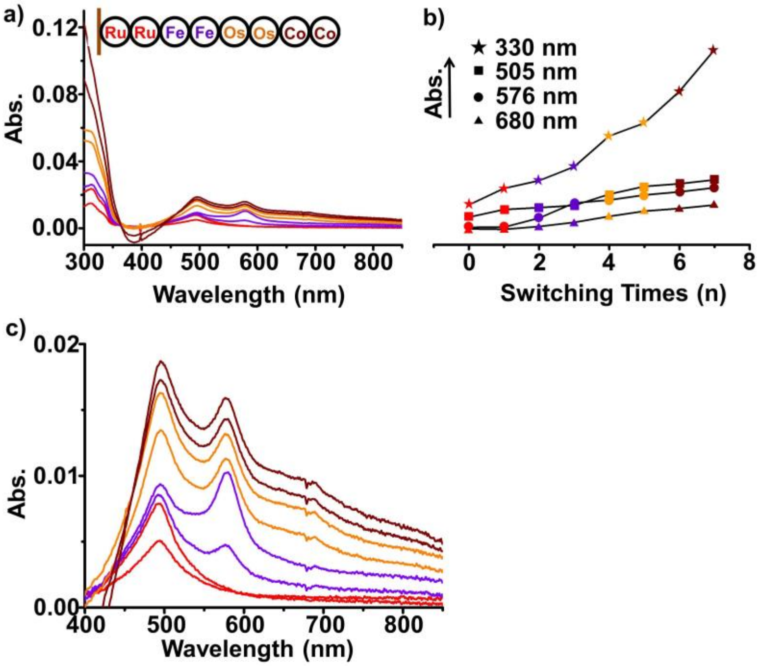

Supplementary Fig. 30 Electrosynthesis of organometallic polymers. a, b Illustrations of UV-vis absorbance and intensities as function of switching times during iterative synthesis. **c** Enlarged view of **a**.

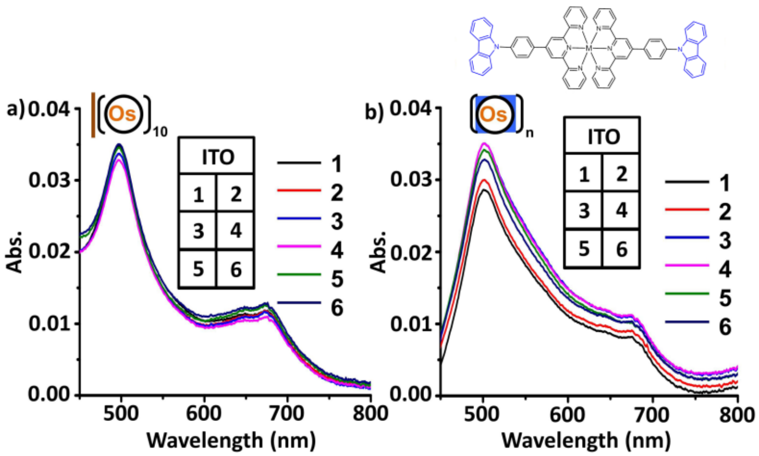

Supplementary Fig. 31 Comparison between assembled polymer film and electropolymerization film. a UV–vis absorption spectra at different positions of self-assembled Os^{II} organometallic polymers on ITO glass. **b** Random poly$(OsCz_2)_n$ from Os complex with two carbazoles and without alkyl unit (M = Os in chemical structure). ITO glass size: 2.0 cm × 4.0 cm. Absorption intensities at different positions have 6% and 18% changes for surface confined organometallic polymers and random polymerization film, respectively, implying that this strategy has superior advantage for fabrication of ultrathin and uniform film in contrast to conventional electropolymerization. Generally, the potential drop on different positions on surface of electrode usually leads to thickness difference for conventional electropolymerization. In this paper, single-monomer-precise is not really affected by potential drop. Additionally, we want to note that the intensity changes in both absorption spectra should be tinily affected by quality of ITO glasses.

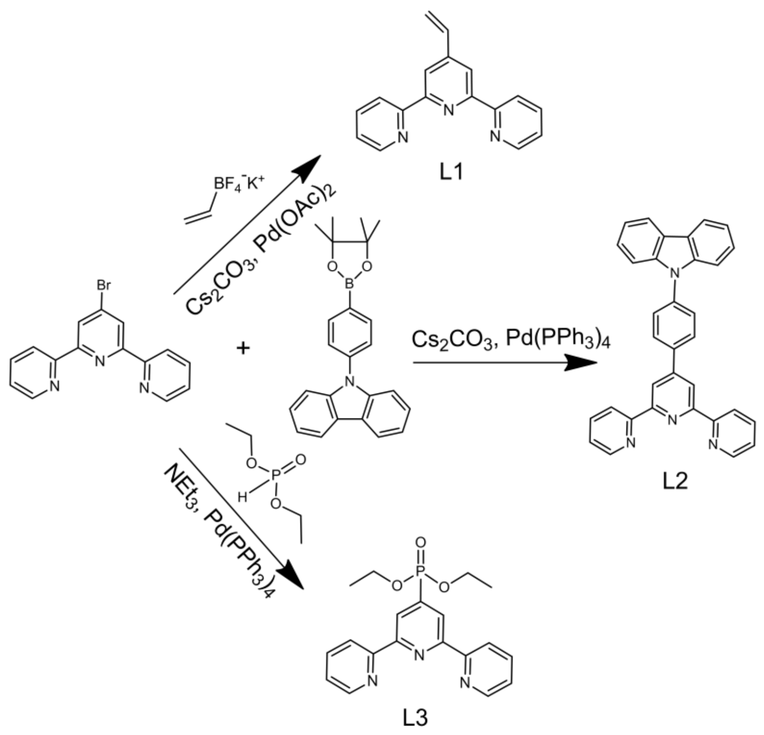

Supplementary Fig. 32 Synthesis. Synthesis routes of the ligands L1, L2 and L3.

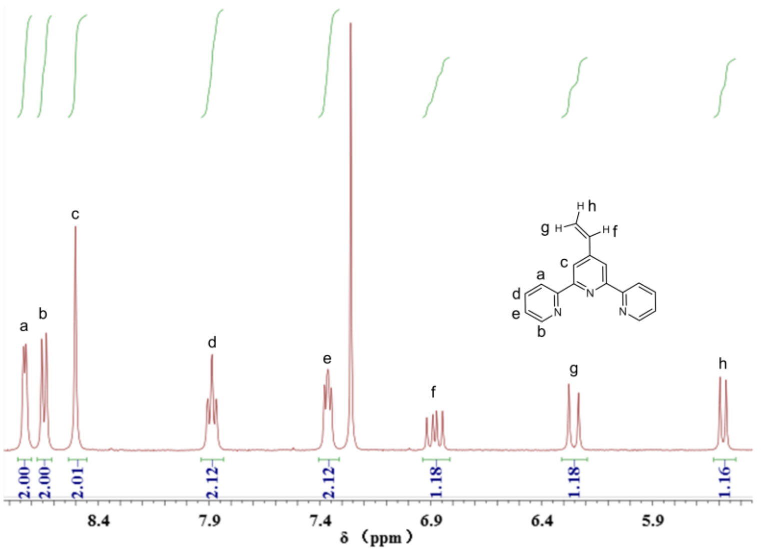

Supplementary Fig. 33 Structural characterization. ^{1}H NMR spectrum of L1 in $CDCl_3$.

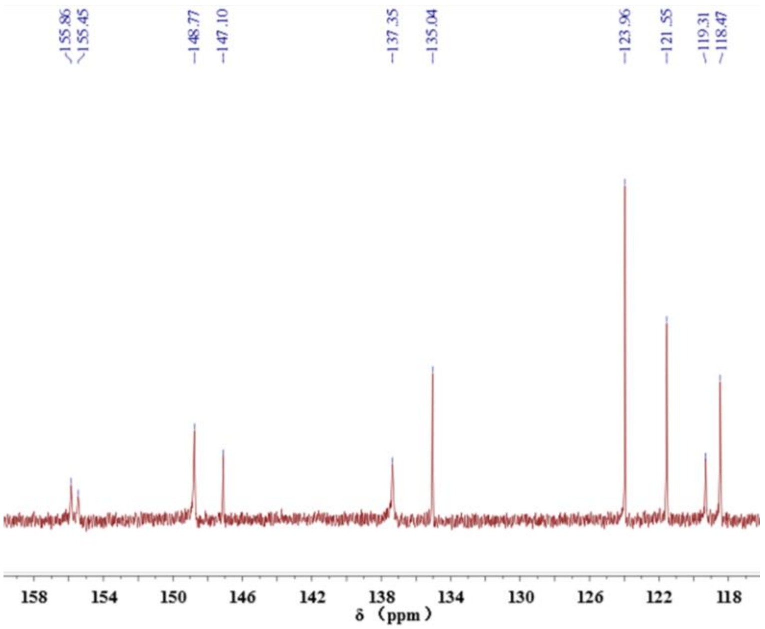

Supplementary Fig. 34 Structural characterization. ^{13}C NMR spectrum of L1 in $CDCl_3$.

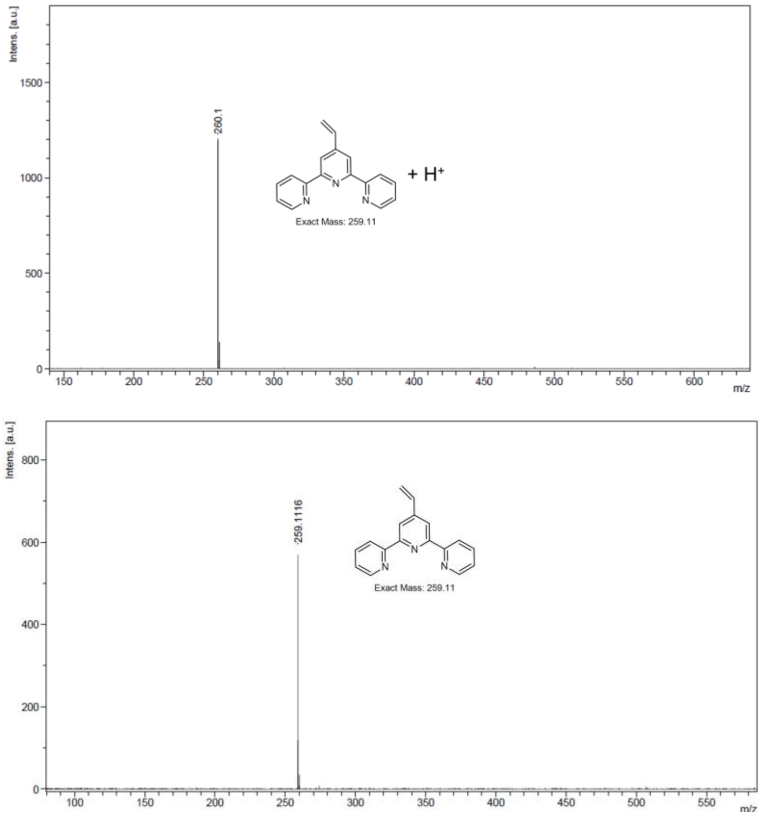

Supplementary Fig. 35 Structural characterization. MALDI-TOF mass spectrum of L1 in $CHCl_3$.

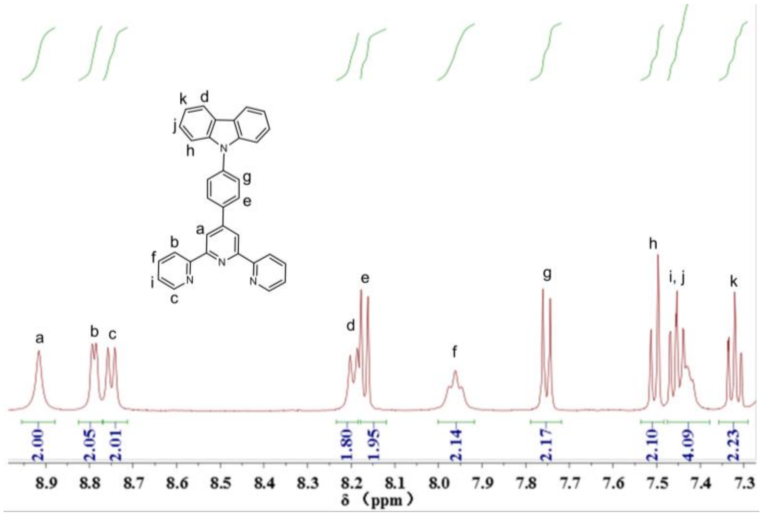

Supplementary Fig. 36 Structural characterization. ^{1}H NMR spectrum of L2 in $CDCl_3$.

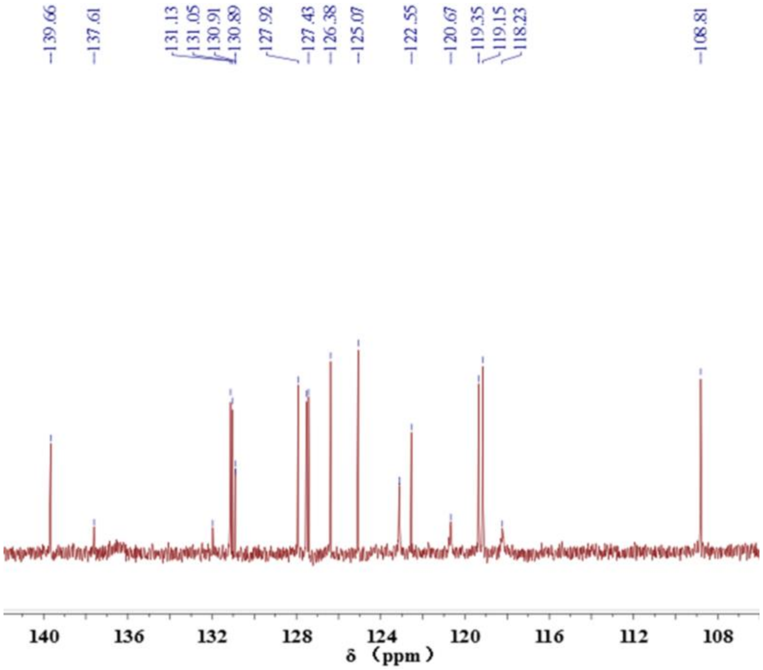

Supplementary Fig. 37 Structural characterization. ^{13}C NMR spectrum of L2 in $CDCl_3$.

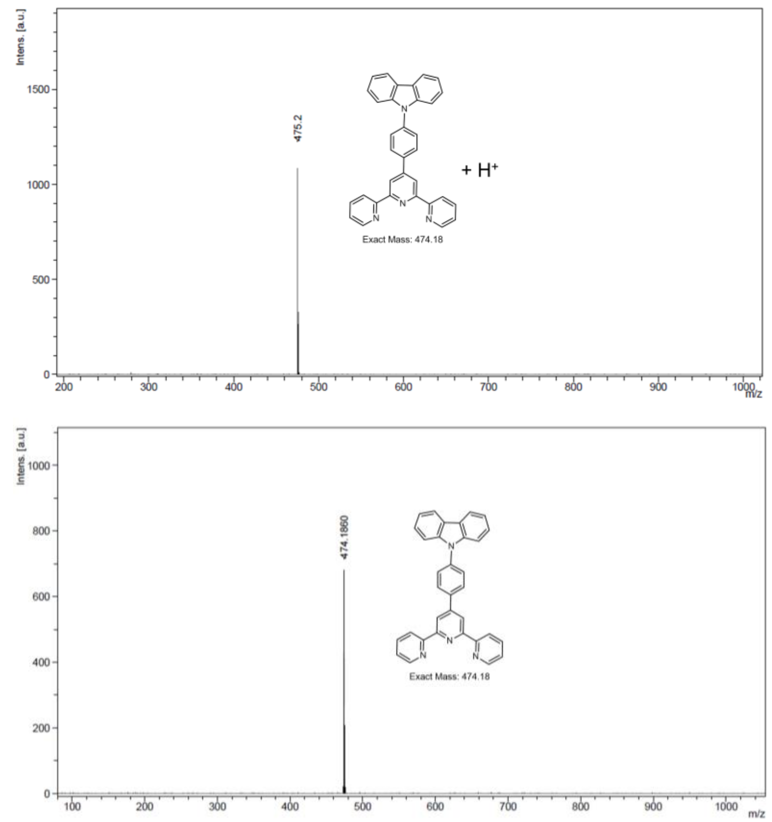

Supplementary Fig. 38 Structural characterization. MALDI-TOF mass spectrum of L2 in $CHCl_3$.

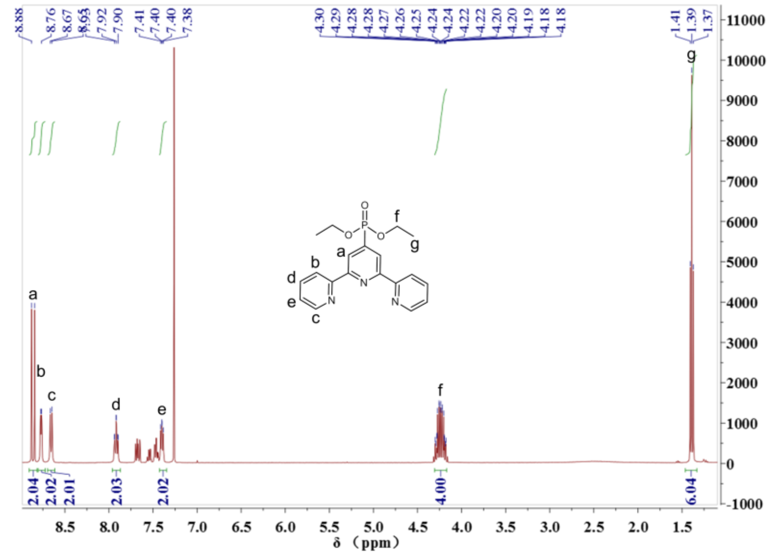

Supplementary Fig. 39 Structural characterization. ^{1}H NMR spectrum of L3 in $CDCl_3$.

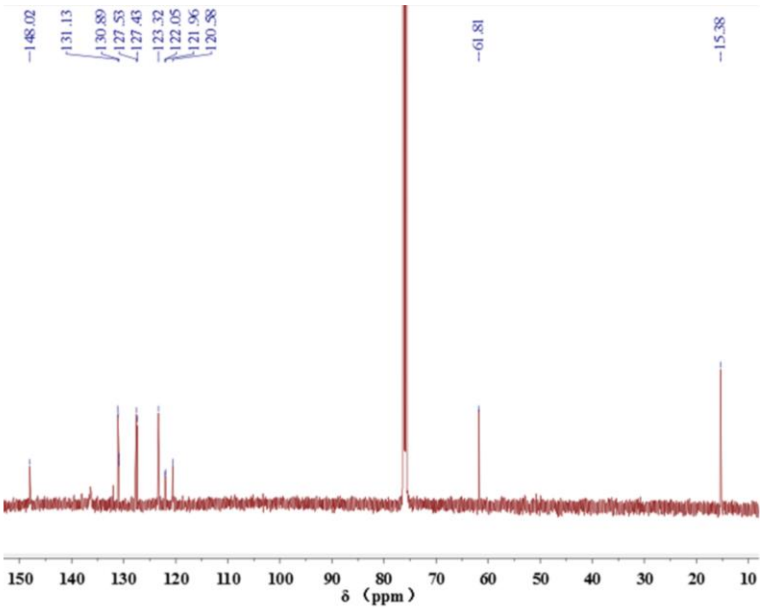

Supplementary Fig. 40 Structural characterization. ^{13}C NMR spectrum of L3 in $CDCl_3$.

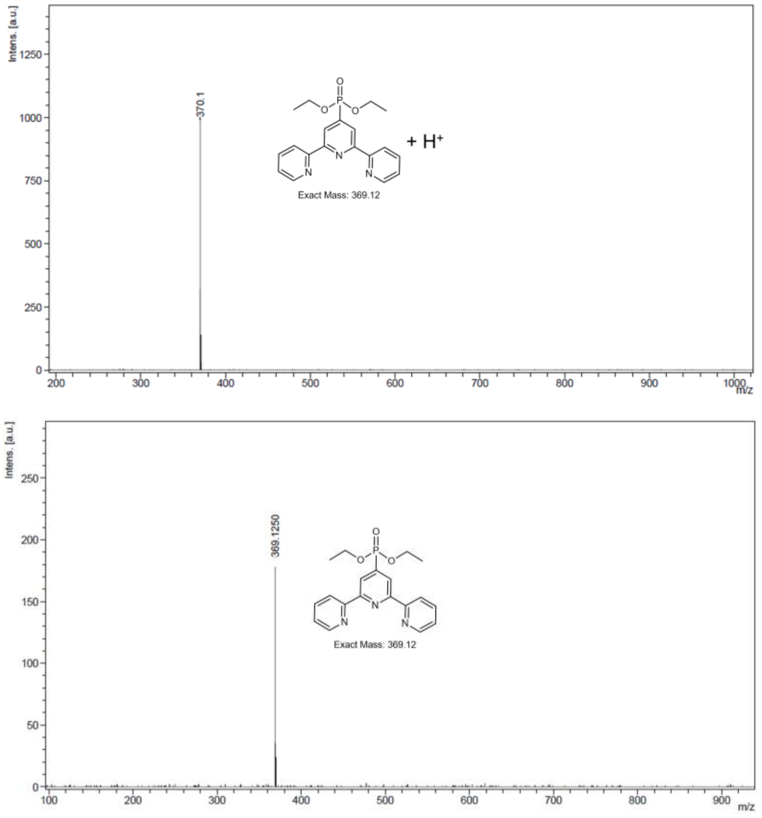

Supplementary Fig. 41 Structural characterization. MALDI-TOF mass spectrum of L3 in $CHCl_3$.

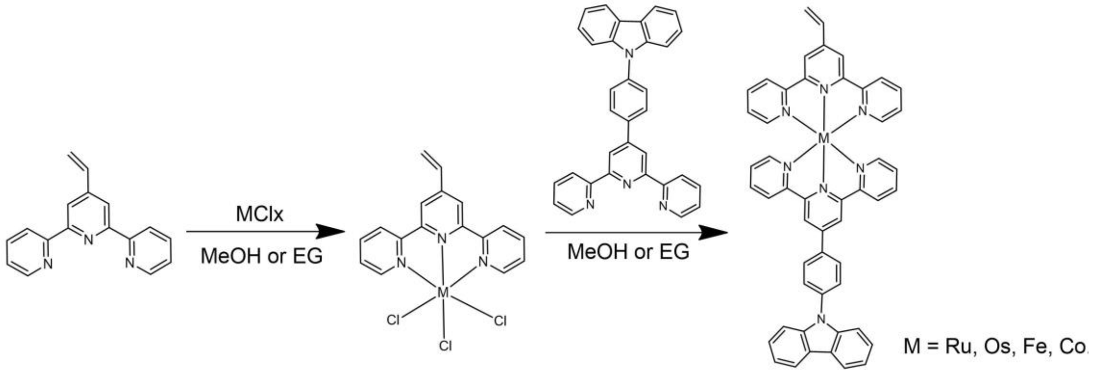

Supplementary Fig. 42 Synthesis. General synthesis routes of the monomers.

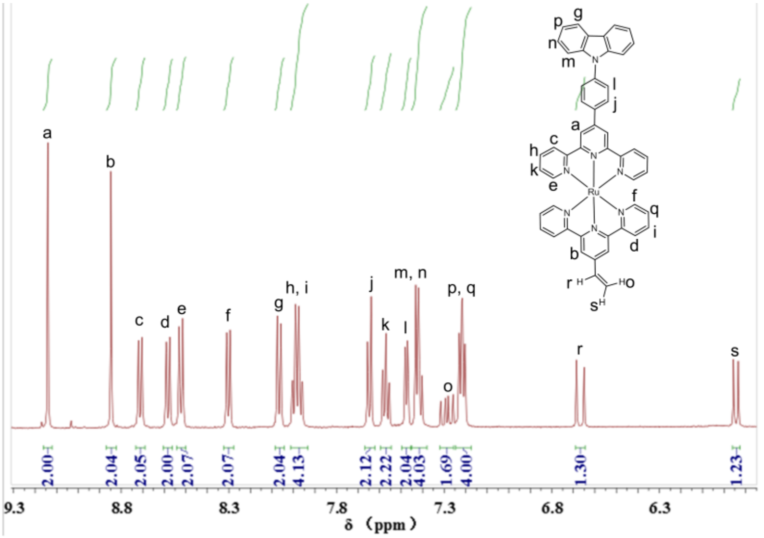

Supplementary Fig. 43 Structural characterization. ^{1}H NMR spectrum of Ru^{II}XY in CD_3CN.

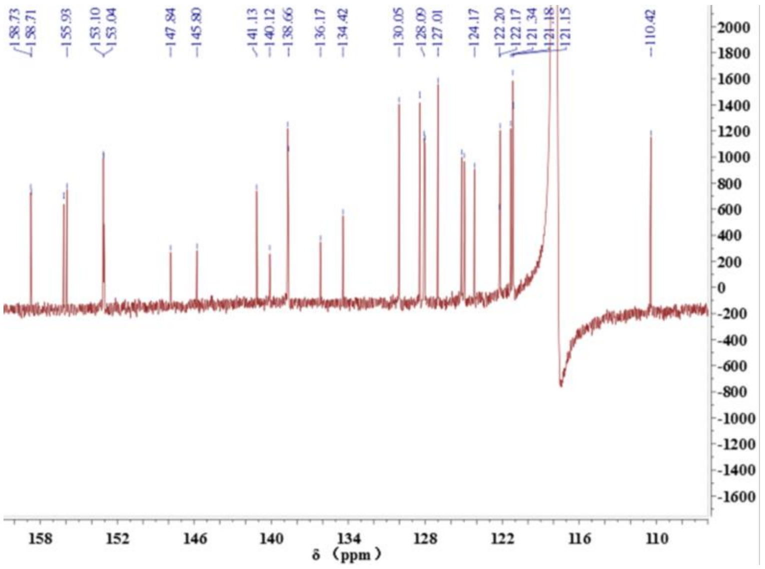

Supplementary Fig. 44 Structural characterization. ^{13}C NMR spectrum of Ru^{II}XY in CD_3CN.

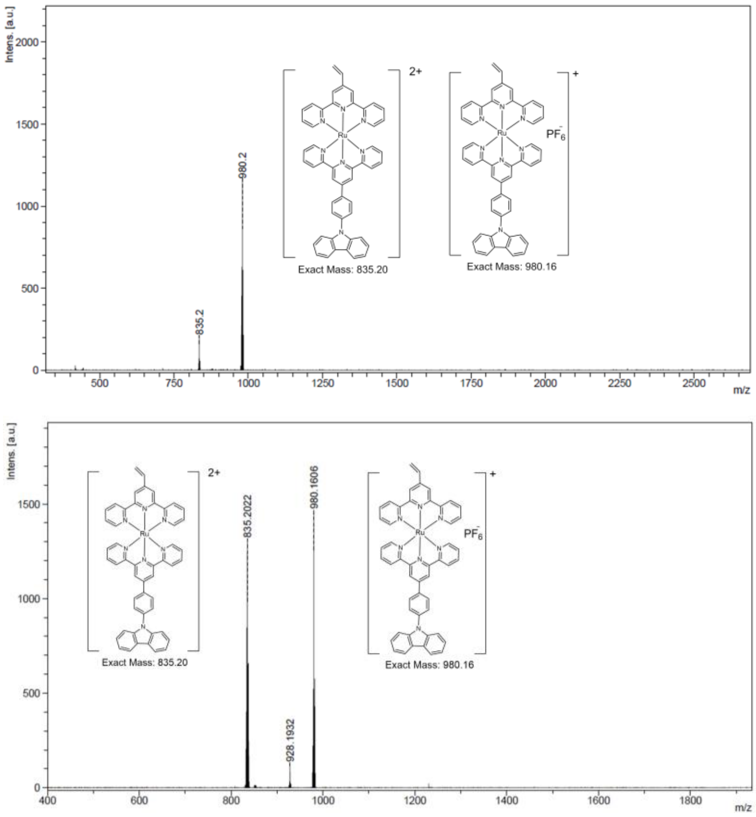

Supplementary Fig. 45 Structural characterization. MALDI-TOF mass spectrum of Ru^{II}XY in CH_3CN.

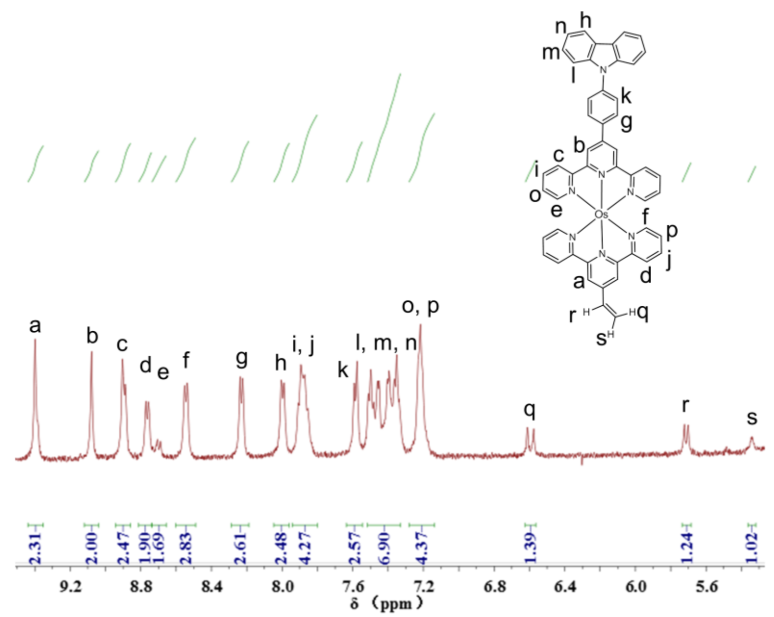

Supplementary Fig. 46 Structural characterization. ^{1}H NMR spectrum of $Os^{II}XY$ in CD_3OD.

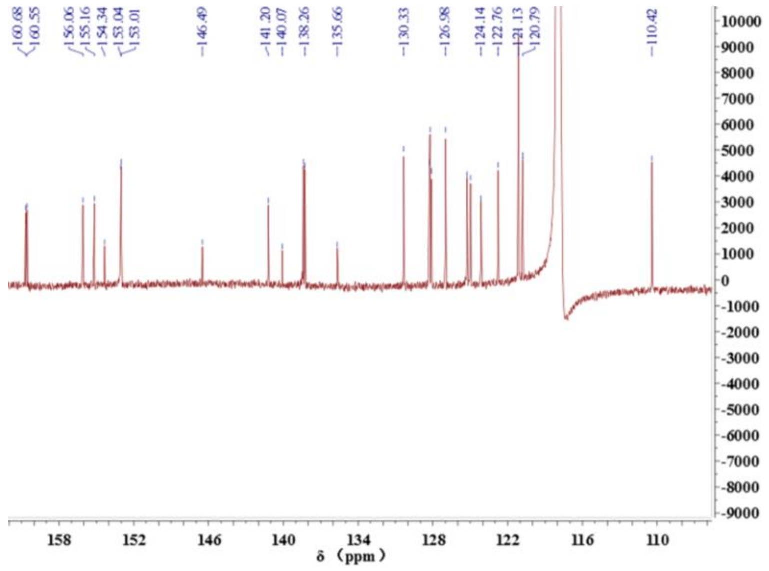

Supplementary Fig. 47 Structural characterization. ^{13}C NMR spectrum of $Os^{II}XY$ in CD_3CN.

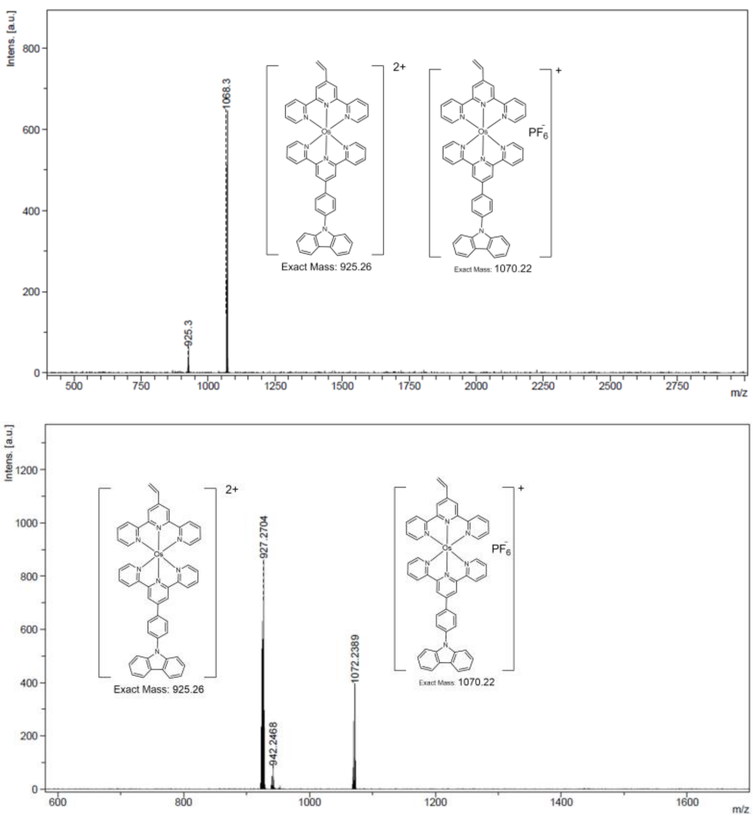

Supplementary Fig. 48 Structural characterization. MALDI-TOF mass spectrum of $Os^{II}XY$ in CH_3CN.

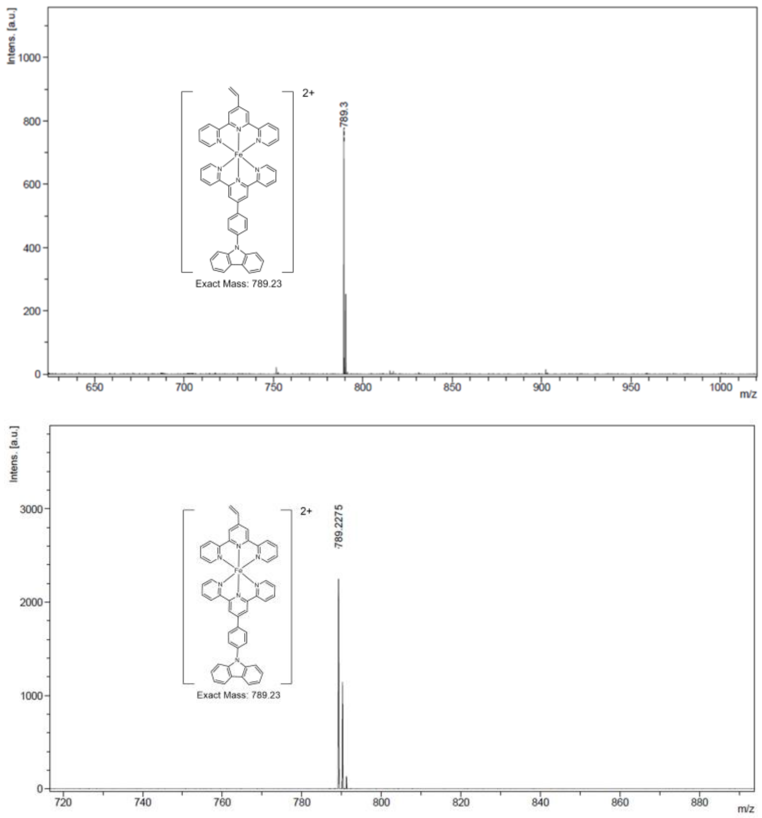

Supplementary Fig. 49 Structural characterization. MALDI-TOF mass spectrum of $Fe^{II}XY$ in CH_3CN.

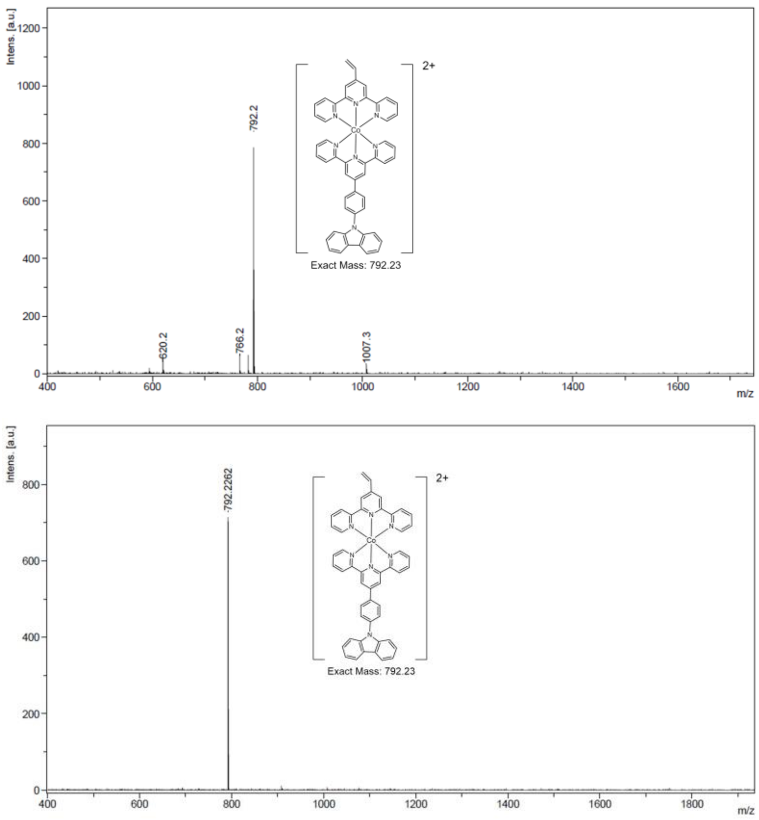

Supplementary Fig. 50 Structural characterization. MALDI-TOF mass spectrum of $Co^{II}XY$ in CH_3CN.

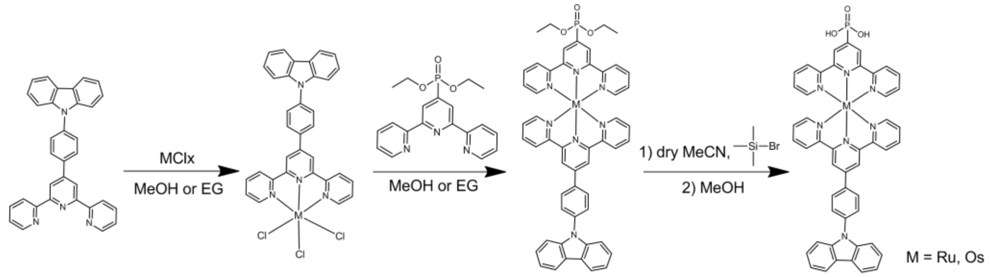

Supplementary Fig. 51 Structural characterization. General synthesis routes of $M^{II}XP$.

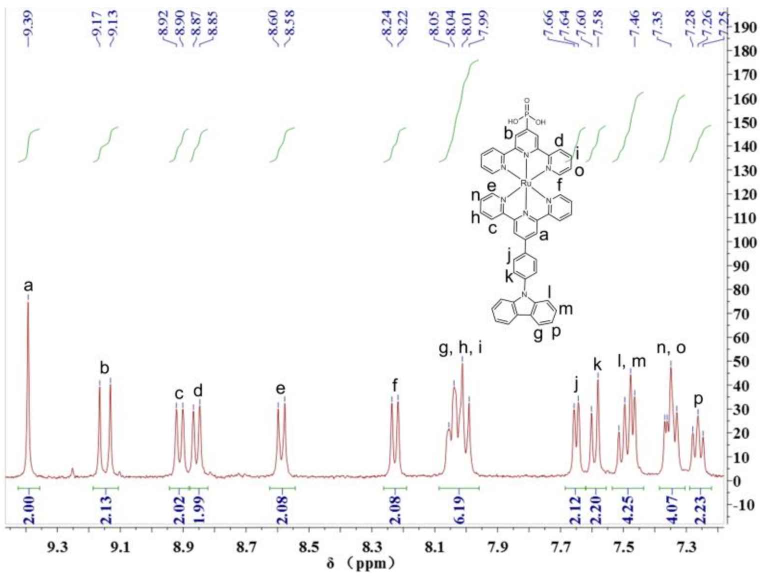

Supplementary Fig. 52 Structural characterization. ^{1}H NMR spectrum of Ru^{II}XP in CD_3OD.

13C NMR spectrum of Ru^{II}XP in CD_3CN.

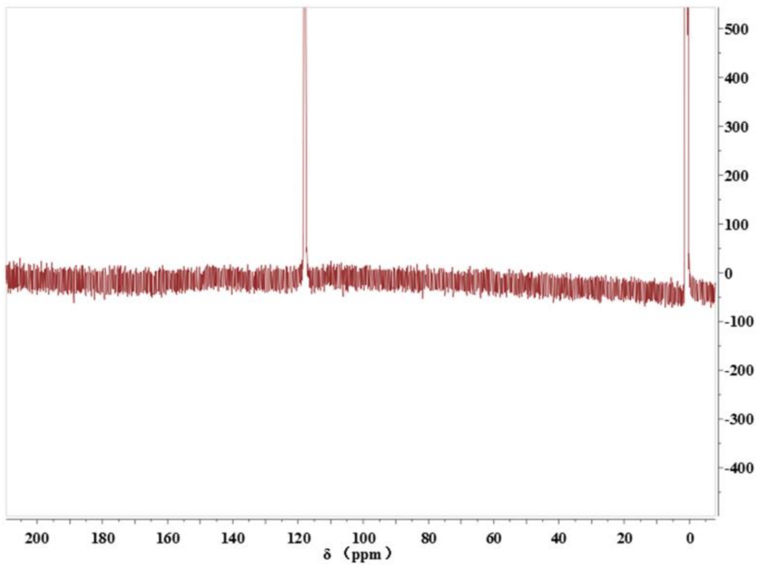

Supplementary Fig. 53 Structural characterization. ^{13}C NMR spectrum of Ru^{II}XP in CD_3CN. We did not get good ^{13}C NMR spectrum because of low solubility.

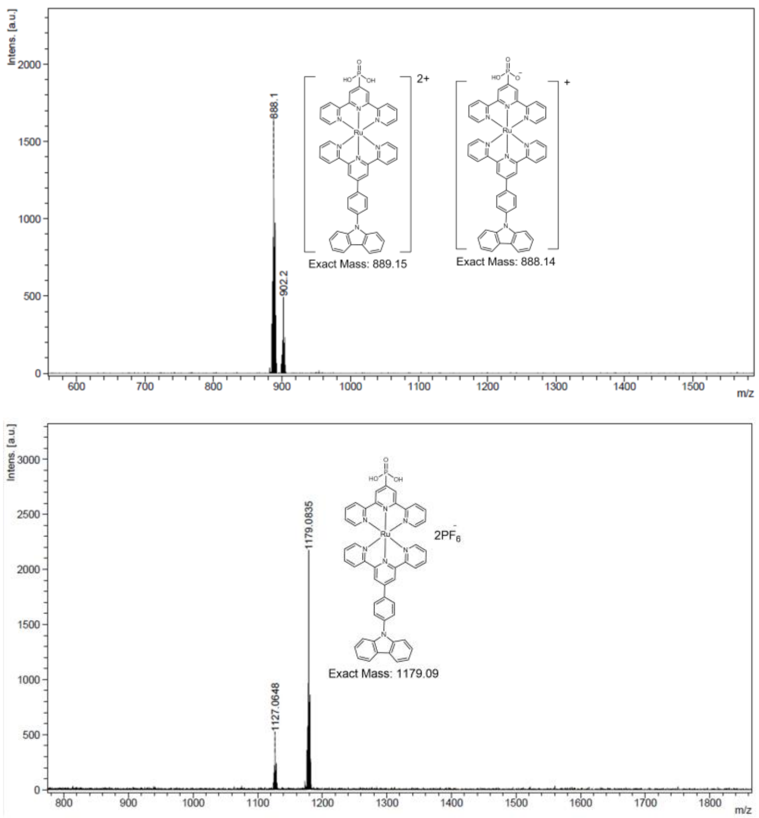

Supplementary Fig. 54 Structural characterization. MALDI-TOF mass spectrum of Ru^{II}XP in CH_3CN.

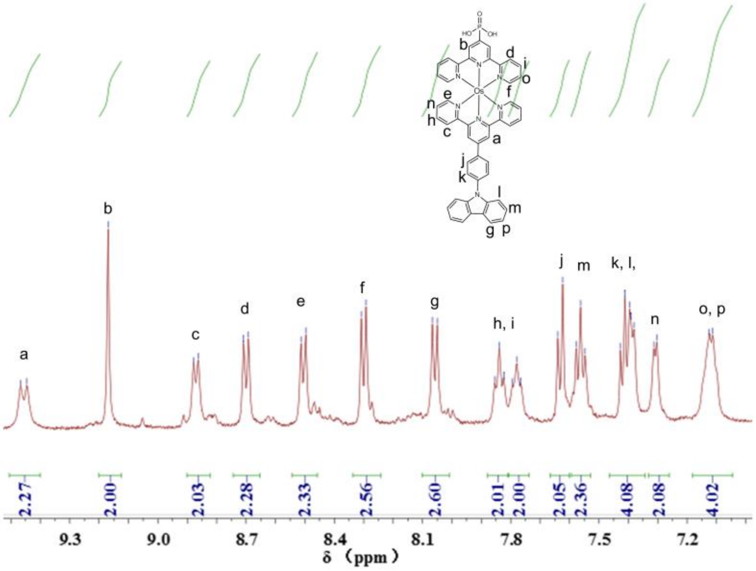

Supplementary Fig. 55 Structural characterization. ^{1}H NMR spectrum of Os^{II}XP in CD_3OD.

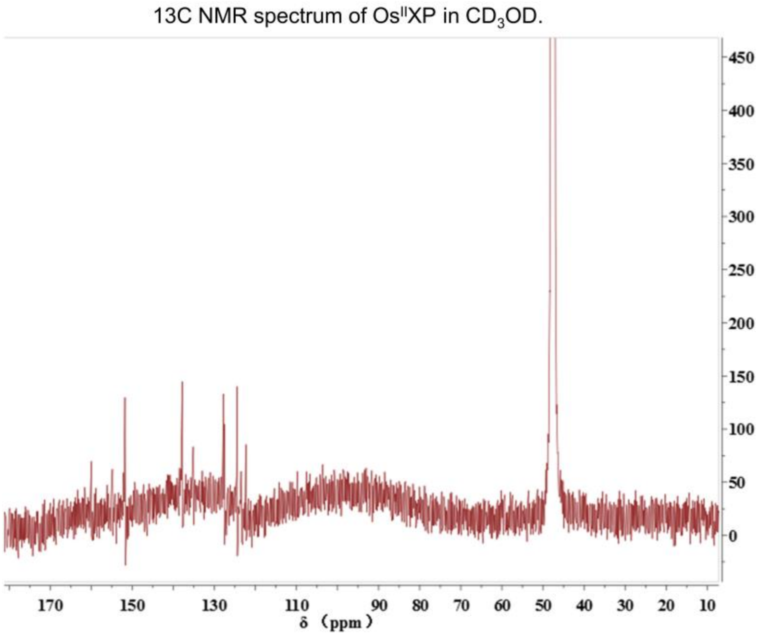

Supplementary Fig. 56 Structural characterization. ^{13}C NMR spectrum of Os^{II}XP in CD_3CN. We did not get good ^{13}C NMR spectrum because of low solubility.

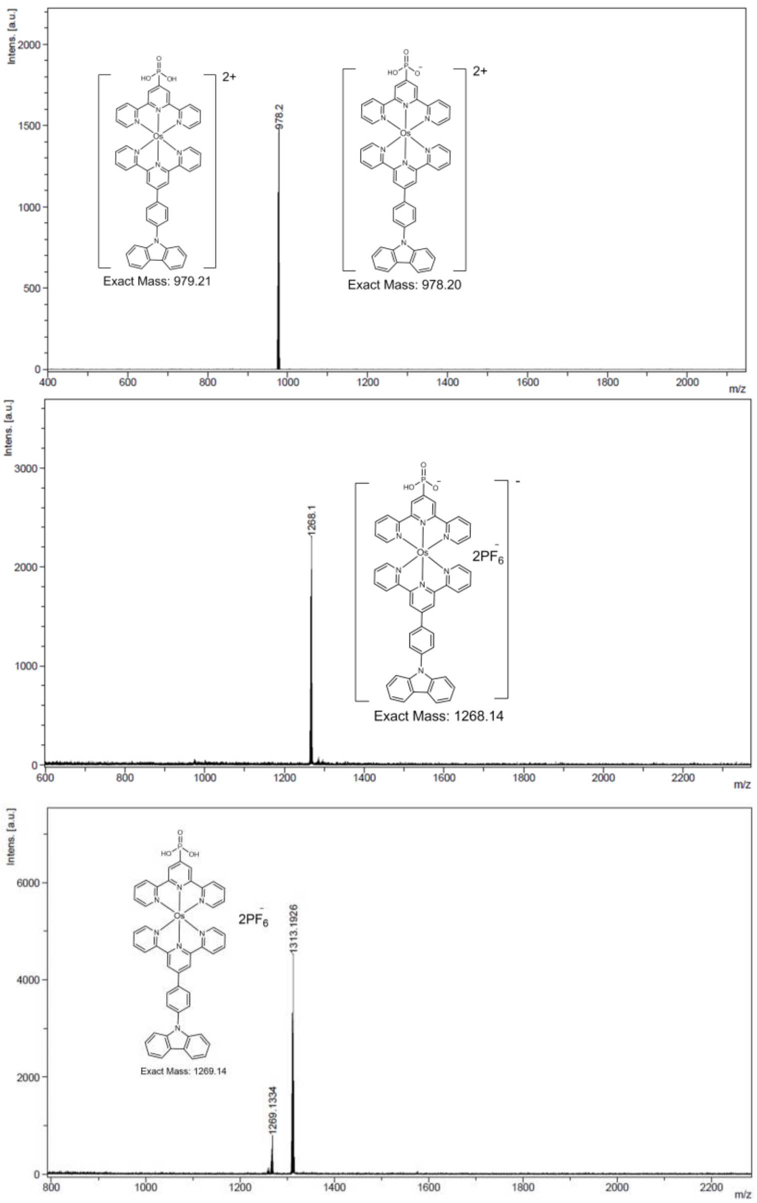

Supplementary Fig. 57 Structural characterization. MALDI-TOF mass spectra of $Os^{II}XP$ in CH_3CN.

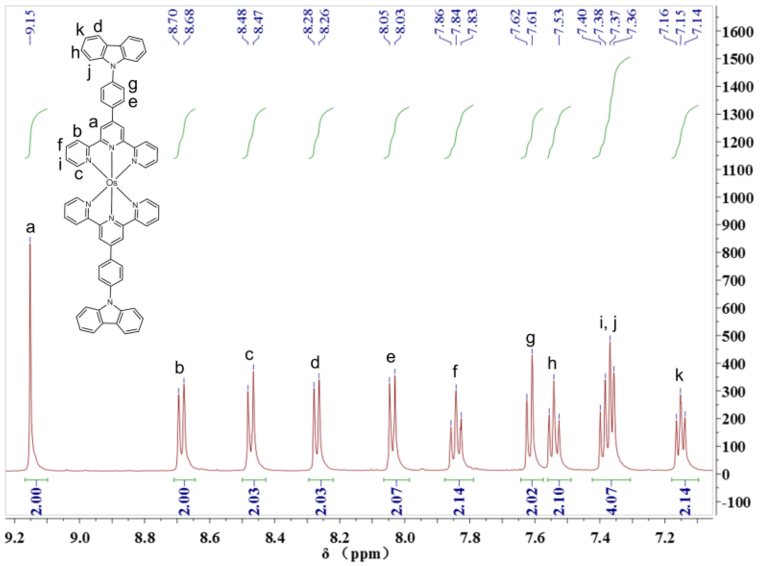

Supplementary Fig. 58 Structural characterization. ^{1}H NMR spectrum of $Os^{II}X_2$ in CD_3CN.

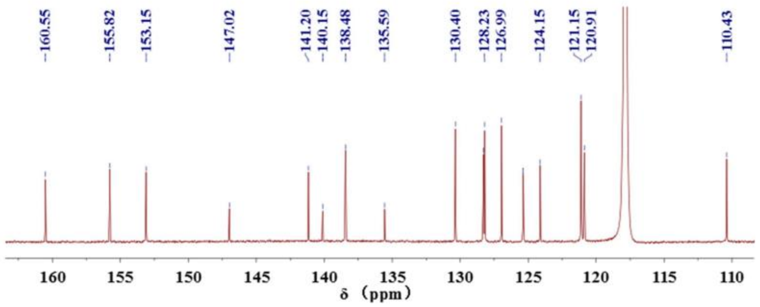

Supplementary Fig. 59 Structural characterization. ^{13}C NMR spectrum of $Os^{II}X_2$ in CD_3CN.

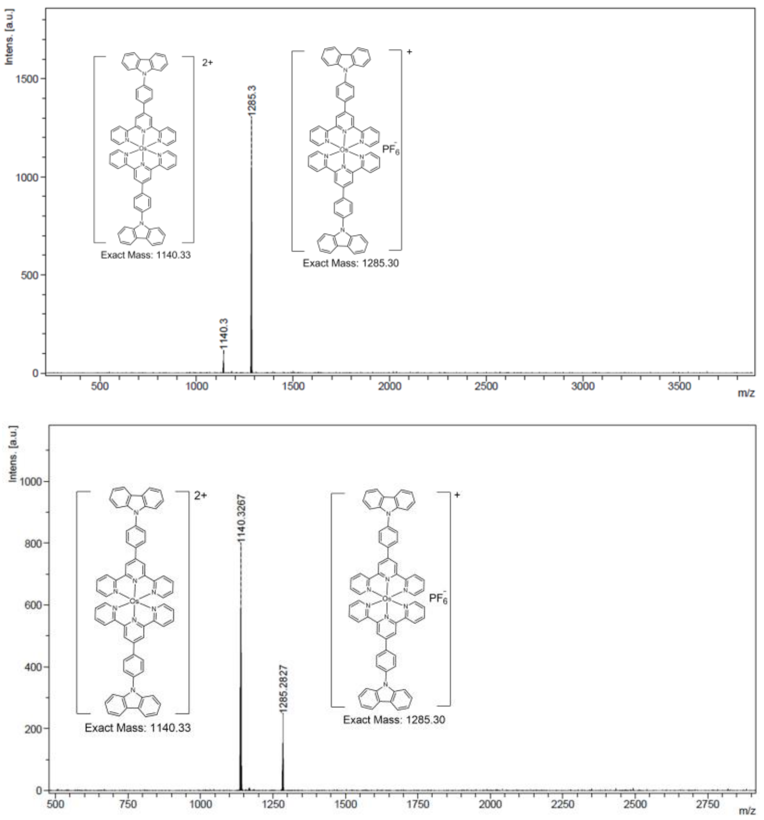

Supplementary Fig. 60 Structural characterization. MALDI-TOF mass spectrum of $Os^{II}X_2$ in CH_3CN.

Supplementary Methods

General Characterizations

^{1}H NMR spectra were obtained at room temperature using a Bruker Avance 300 NMR spectrometer. Electronic absorption spectra were recorded on a JASCO V-770 spectrophotometer at room temperature. Photoluminescence spectra were recorded using Perkin Elmer LS50B Luminescence Spectrometer. AFM study was carried out using a commercial AFM unit (SPA 300HV with a SPI 3800N Probe Station, Seiko Instruments Inc., Japan). All AFM images were taken in dynamic force mode (DFM, i.e., tapping mode) at optimal force. SEM images were obtained using a FEL XL30ESEM-FEG scanning electron microscope with acceleration voltage of 10-20 keV. The atomic structure of the Os^{II} and Ru^{II} alternative organometallic polymers was characterized using an ARM-200CF (JEOL, Tokyo, Japan) scanning transmission electron microscope

(STEM) operated at 200 keV and equipped with double spherical aberration (Cs) corrector. The attainable resolution of the probe defined by the objective pre-field is 78 picometers. MALDI-TOF and ESI MS were obtained using Bruker Daltonics Autoflex III TOF, but the mass spectra on ITO surface were obtained using Bruker UltrafleXtreme in Univ. South Florida, USA.

Synthesis of 4'-vinyl-2,2':6',2''-terpyridine (L1)

L1 was successfully synthesized using the method reported by Zhong's group.[6] Specifically, a suspension of 4'-bromoterpyridine (200 mg, 0.640 mM), potassium vinyltrifluoroborate (104 mg, 0.760 mM, 1.20 equiv), $Pd(OAc)_2$ (3.0 mg, 0.0128 mM), PPh_3 (10.0 mg, 0.0400 mM), and Cs_2CO_3 (652 mg, 2.00 mM) in THF/H_2O (9.6 mL / 0.4 mL) was heated at 85 ℃ under argon atmosphere in a sealed flask. After 48 h, the reaction was quenched by adding 10 mL of H_2O and allowed to cool down to room temperature, followed by extraction with CH_2Cl_2 (30 mL×3). The organic phases were combined, and washed with brine and dried with Na_2SO_4. The solvent was removed under reduced pressure, and the crude product was purified by silica gel chromatography (eluting with 65:65:1 petroleum ether/CH_2Cl_2/NH_4OH) to yield L1 as a white solid (133 mg, 80.0%). ^{1}H NMR (400 MHz, $CDCl_3$) δ 8.73 (d, J = 4.1 Hz, 2H), 8.64 (d, J = 7.9 Hz, 2H), 8.50 (s, 2H), 7.89 (t, J = 7.7, 2H), 7.41 – 7.31 (m, 2H), 6.88 (dd, J = 17.6, 10.9 Hz, 1H), 6.26 (d, J = 17.6 Hz, 1H), 5.58 (d, J = 10.9 Hz, 1H).

Synthesis of 9-(4-([2,2':6',2''-terpyridin]-4'-yl)phenyl)-9H-carbazole (L2)

L2 was synthesized using the classical protocol of Suzuki-Miyaura cross-coupling reaction: A suspension of 4'-bromoterpyridine (366 mg, 1.17 mM), Na_2CO_3 (1.27 g, 12.0 mM), 9-(4-(4,4,5,5-tetramethyl-1,3,2-dioxaborolan-2-yl)phenyl)-9H-carbazole (394 mg, 1.07 mM), $Pd(PPh_3)_4$ (61.8 mg, 0.0535 mM) in toluene/H_2O (9 mL / 6 mL) was heated at 90 ^{o}C under argon atmosphere in a sealed flask. After 24 h, the reaction was quenched by adding 10 mL of H_2O and allowed to cool down to room temperature, followed by extraction with CH_2Cl_2 (30 mL×3). The organic phases were combined, and washed with

brine and dried with Na_2SO_4. The solvent was removed under reduced pressure, and the crude product was purified by silica gel chromatography (eluting with 65:65:1 petroleum ether/CH_2Cl_2/NH_4OH) to yield L2 as a white solid (370 mg, 73.1%). ^{1}H NMR (400 MHz, $CDCl_3$) δ 8.92 (s, 2H), 8.79 (d, J = 4.2 Hz, 2H), 8.75 (d, J = 7.9 Hz, 2H), 8.19 (d, J = 8.3 Hz, 2H), 8.17 (d, J = 7.8 Hz, 2H), 7.96 (t, J = 7.0 Hz, 2H), 7.75 (d, J = 8.4 Hz, 2H), 7.50 (d, J = 8.2 Hz, 2H), 7.48–7.40 (m, 4H), 7.32 (t, J = 7.4 Hz, 2H).

Synthesis of Diethyl [2,2':6',2''-terpyridin]-4'-ylphosphonate (L3)

L3 was synthesized according to previous report.[7] In detail, a suspension of 4'-bromoterpyridine (312 mg, 1.00 mM), diethyl phosphite (414 mg, 3.00 mM), $Pd(PPh_3)_4$ (57.8 mg, 0.0500 mM) and triethylamine (0.3 mL, 2.00 mM) in toluene (10 mL) was heated at 95 ^oC under argon atmosphere in a sealed flask equipped with a spherical condenser. After 12 h, the reaction was quenched by adding 10 mL of H_2O, and allowed to cool down to room temperature, followed by extraction with CH_2Cl_2 (30 mL×3). The organic phases were combined and washed with brine and dried with Na_2SO_4. The solvent was removed under reduced pressure, and the crude product was purified by silica gel chromatography (eluting with 5:1 CH_2Cl_2/MeOH) to yield L3 as a white solid (133 mg, 80.0%). ^{1}H NMR (400 MHz, $CDCl_3$) δ 8.86 (d, *J* = 13.8 Hz, 2H), 8.77 (d, *J* = 4.2 Hz, 2H), 8.66 (d, *J* = 7.9 Hz, 2H), 7.92 (td, *J* = 7.8, 1.4 Hz, 2H), 7.40 (dd, *J* = 6.6, 5.0 Hz, 2H), 4.31 – 4.17 (m, 4H), 1.39 (t, *J* = 7.1 Hz, 6H); ^{31}P NMR (400 MHz, $CDCl_3$): δ 14.99.

Synthesis of Ru^{II}XY

A two-step coordination protocol was applied to synthesize asymmetric Ru^{II}XY type of complexes. Firstly, $RuCl_3$•$3H_2O$ (52.2 mg, 0.200 mM) and equal molar L1 (51.8 mg, 0.200 mM) in MeOH (5 mL) were introduced into a round bottom flask equipped with a condenser. This mixture was heated to reflux and allowed to reaction at that temperature for 4 h. The single coordination intermediate $RuL1Cl_3$ precipitated in mixture was isolated and collected by simply filtering process. The dark red precipitation was washed

with H_2O and diethyl ether, and dried in vacuum oven, and it allowed to react with L2 (94.8 mg, 0.200 mM) in refluxed MeOH solution for another 12 h. The solvent was removed under reduced pressure, and the crude product was purified by silica gel chromatography (eluting with 30:1:0.05 CH_3CN/H_2O/saturated KNO_3 aq.). The exchange of counterion was completed by adding saturated NH_4PF_6 aq., the precipitation was filtered, washed with H_2O, diethyl ether and dried to yield $Ru^{II}XY$ as red powder (118 mg, 60.2%). ^1H NMR (500 MHz, CD_3CN) δ 9.14 (s, 2H), 8.85 (s, 2H), 8.71 (d, *J* = 8.0 Hz, 2H), 8.58 (d, *J* = 8.0 Hz, 2H), 8.52 (d, *J* = 8.5 Hz, 2H), 8.30 (d, *J* = 7.8 Hz, 2H), 8.07 (d, *J* = 8.4 Hz, 2H), 7.98 (td, *J* = 8.5, 1.3 Hz, 4H), 7.65 (d, *J* = 8.2 Hz, 2H), 7.60 – 7.55 (m, 2H), 7.48 (d, *J* = 5.1 Hz, 2H), 7.45 – 7.38 (m, 4H), 7.29 (dd, *J* = 17.6, 11.0 Hz, 1H), 7.22 (dd, *J* = 7.3, 5.9 Hz, 4H), 6.67 (d, *J* = 17.6 Hz, 1H), 5.95 (d, *J* = 11.0 Hz, 1H).

Synthesis of $Os^{II}XY$

$Os^{II}XY$ was synthesized by stepwise coordinaiton of $(NH_4)_2OsCl_6$ (43.9 mg, 0.100 mM) with equal molar L1 (25.9 mg, 0.100 mM) and L2 (47.4 mg, 0.100 mM) in refluxed ethylene glycol (EG) solution succesively. The crude product was purified by silica gel chromatography with an eluent of CH_3CN/toluene (1:1, volume ratio) mixed solution, and counterion exchange was completed by adding saturated NH_4PF_6 aq., and the precipitation was filtered, washed and dried to yield $Os^{II}XY$ as brown powder (60.8 mg, 50.3%). ^1H NMR (500 MHz, CD_3OD) δ 9.40 (s, 2H), 9.08 (s, 2H), 8.90 (d, *J* = 6.5 Hz, 2H), 8.76 (d, *J* = 7.0 Hz, 1H), 8.70 (d, *J* = 9.4 Hz, 2H), 8.54 (d, *J* = 8.0 Hz, 3H), 8.23 (dd, *J* = 7.1, 0.8 Hz, 3H), 8.05 – 7.96 (m, 3H), 7.88 (dd, *J* = 17.9, 8.4 Hz, 4H), 7.58 (d, *J* = 7.3 Hz, 3H), 7.52 – 7.33 (m, 3H), 7.28 – 7.14 (m, 4H), 6.59 (d, *J* = 17.0 Hz, 1H), 5.71 (d, *J* = 10.7 Hz, 1H), 5.34 (d, *J* = 3.9 Hz, 1H).

Synthesis of $Fe^{II}XY$

$FeCl_3$ (32.2 mg, 0.200 mM) was dissolved in 10 mL MeOH in a Schlenk flask, methanol solution of L1 (51.8 mg, 0.200 mM) was dropwise introduced via a syringe under vigorous stiring in room temperature. This mixture was allowed to react in room

temperature for 1 h. The coordination intermediate $FeL1Cl_3$ precipitated in mixture was isolated and collected by filtering, washing with diethyl ether and drying in vacuum oven. The yellow precipitation (42.0 mg, 0.100 mM) then allowed to react with L2 (47.4 mg, 0.100 mM) in refluxed MeOH solution for another 12 h. The resultant mixture was concentrated under reduced pressure, and the crude product was purified by silica gel chromatography with an eluent of CH_3CN/toluene (3:1, volume ratio) mixed solution. Then saturated NH_4PF_6 aq. was added to complete the counterion exchange, the precipitation was filtered, washed and dried to yield $Fe^{II}XY$ as purple powder (48.6 mg, 45.0%).

Synthesis of $Co^{II}XY$

$CoCl_2$ (39.0 mg, 0.300 mM) was dissolved in 10 mL MeOH in a Schlenk flask equiped with a condenser, the system was then sealed and heated to 40 ^oC under argon atmosphere, methanol solution of L2 (142 mg, 0.300 mM) was dropwise added via a syringe under vigorous stiring, then heated up to reflux and reacted for 1 h. The coordination intermediate (143 mg, 0.240 mM) was retrieved as a green precipitation by filtering, washing with diethyl ether and drying in vacuum oven, then allowed to react with L1 (61.5 mg, 0.240 mM) in refluxed MeOH solution for another 12 h. The resultant mixture was concentrated under reduced pressure, and the crude product was purified by silica gel chromatography with an eluent of CH_3CN/toluene (3:1, volume ratio) mixed solution. Then saturated NH_4PF_6 aq. was added to complete the counterion exchange, and the precipitation was filtered, washed and dried to yield $Co^{II}XY$ as pale orange powder (77.0 mg, 30.2%).

Synthesis of $Ru^{II}XP$

$RuCl_3 \cdot 3H_2O$ (52.2 mg, 0.200 mM) and L2 (94.8 mg, 0.200 mM) dissolving in 10 mL MeOH were loaded into a round bottom flask equipped with a condenser, the mixture was heated to reflux under argon atmosphere, reacted at that temperature for 4 h. The single coordination intermediate $RuL2Cl_3$ precipitated in mixture, it was isolated and

collected by filtering, washing with H_2O, diethyl ether and drying. Then $RuL2Cl_3$ was subjected to react with L3 (73.8 mg, 0.200 mM) in refluxed MeOH solution for another 12 h. The resultant mixture was concentrated under reduced pressure, and the product was precipitated in the mixture by adding saturated NH_4PF_6 aq., and then isolated, and collected by filtering, washing and drying steps. The crude product was further subjected to a two-step alcoholysis process to obtain the target molecule Ru^{II}XP according to reported method.[8] In detail, complex RuL2L3 (36.0 mg, 0.029 mM) dissolving in 10 mL anhydrous CH_3CN was loaded into a dried three-neck round bottom flask, which was equipped with a condenser. The system was then sealed, and trimethylbromosilane (0.100 mL, 0.758 mM) was introduced via a syringe under argon atmosphere. This mixture was left to react at 70 ^{o}C for 48 h. After cooling down to room temperature, 1.0 mL methanol was injected into the mixture via a syringe, and stirred for another 1 h to complete the alcoholysis. The solvent was evaporated under reduced pressure, a small amout of methanol was added for just dissolving the crude product, saturated NH_4PF_6 aq. was then added for counterion exchange. The resultant precipitation was filtered, washed with diethyl ether and dried to yield Ru^{II}XP as a red powder (25.3 mg, 74.2%). ^{1}H NMR (400 MHz, CD_3OD) δ 9.39 (s, 2H), 9.15 (d, *J* = 13.6 Hz, 1H), 8.91 (d, *J* = 8.0 Hz, 2H), 8.86 (d, *J* = 8.0 Hz, 2H), 8.59 (d, *J* = 8.4 Hz, 2H), 8.23 (d, *J* = 7.8 Hz, 2H), 8.02 (dd, *J* = 18.1, 7.5 Hz, 6H), 7.65 (d, *J* = 5.4 Hz, 2H), 7.59 (d, *J* = 8.2 Hz, 2H), 7.49 (dd, *J* = 13.6, 6.3 Hz, 2H), 7.35 (dd, *J* = 10.0, 5.3 Hz, 2H), 7.29 – 7.22 (m, 2H).

Synthesis of Os^{II}XP

$(NH_4)_2OsCl_6$ (43.9 mg, 0.100 mM) and L2 (47.4 mg, 0.100 mM) dissolving in 10 mL ethylene glycol were loaded into a round bottom flask equipped with a condenser, the mixture was heated up to reflux and allowed to react at that temperature for 12 h under argon atmosphere. After cooling down, L3 (36.9 mg, 0.100 mM) dissolving in 5.0 mL ethylene glycol was added, and the mixture was left to react in reflux for another 12 h under argon atmosphere. Complex Os^{II}L2L3 was isolated by adding saturated NH_4PF_6

aq., and resultant precipitation was collected by filtering, washing and drying steps. Complex Os^{II}L2L3 (127.0 mg, 0.0958 mM) dissolving in 10.0 mL anhydrous CH_3CN was loaded into a dried three-neck round bottom flask, which was equipped with a condenser. The system was then sealed, and trimethylbromosilane (0.100 mL, 0.758 mM) was introduced via a syringe under argon atmosphere. This mixture was left to react at 70 ^{o}C for 48 h. After cooling down, 1.0 mL methanol was injected into the mixture via a syringe, stirred for another 1 h to complete the alcoholysis. The solvent was evaporated under reduced pressure, then the crude product was purified by silica gel chromatography with an eluent of CH_3CN/CH_3OH/saturated KNO_3 aq. (50:50:1, volume ratio). The product was further purified by adding saturated NH_4PF_6 aq. into its methanol solution, the resultant precipitation was filtered, washed with diethyl ether and dried to yield Os^{II}XP as a dark brown powder (60.8 mg, 50.1%). ^{1}H NMR (500 MHz, CD_3CN) δ 9.46 (d, J = 10.4 Hz, 2H), 9.17 (s, 2H), 8.87 (d, J = 8.5 Hz, 2H), 8.70 (d, J = 8.2 Hz, 2H), 8.51 (d, J = 8.1 Hz, 2H), 8.30 (d, J = 7.9 Hz, 2H), 8.06 (d, J = 8.2 Hz, 2H), 7.88 – 7.81 (m, 2H), 7.81 – 7.74 (m, 2H), 7.63 (d, J = 8.2 Hz, 2H), 7.60 – 7.53 (m, 2H), 7.46 – 7.34 (m, 4H), 7.31 (d, J = 4.9 Hz, 2H), 7.12 (m, J = 6.1 Hz, 4H).

Synthesis of $Os^{II}X_2$

$(NH_4)_2OsCl_6$ (43.9 mg, 0.100 mM) and L2 (94.8 mg, 0.200 mM) dissolving in 10 mL ethylene glycol were loaded into a round bottom flask equipped with a condenser, and the mixture was heated up to reflux and allowed to react at that temperature for 12 h under argon atmosphere. The solvent was evaporated under reduced pressure. The crude product was purified by silica gel chromatography with an eluent of CH_3CN/toluene (1:1, volume ratio). The product was further purified by adding saturated NH_4PF_6 aq. into its methanol solution. The resultant precipitation was filtered, washed with diethyl ether and dried to yield $Os^{II}X_2$ as a dark brown powder (45.1 mg, 63.1%). ^{1}H NMR (500 MHz, CH_3CN+D_2O) δ 9.15 (s, 2H), 8.69 (d, *J* = 8.1 Hz, 2H), 8.47 (d, *J* = 8.3 Hz, 2H), 8.27 (d, *J* = 7.8 Hz, 2H), 8.04 (d, *J* = 8.3 Hz, 2H), 7.84 (t, *J* = 7.8 Hz, 2H), 7.62 (d, *J* = 7.8 Hz, 2H),

7.54 (t, *J* = 7.6 Hz, 2H), 7.38 (dd, *J* = 14.0, 6.8 Hz, 4H), 7.18 – 7.10 (m, 2H).

Supplementary References

1. Xia, C. & Advincula, R. C. Surface grafting of conjugated polymers onto self-assembled monolayer modified conducting substrates by electrochemistry. *Chem. Mater.* **13**, 1682-1691 (2001).

2. Lv, Y., Yao, L., Gu, C., Xu, Y., Liu, D., Lu, D. & Ma, Y. Electroactive self-assembled monolayers for enhanced efficiency and stability of electropolymerized luminescent films and devices. *Adv. Funct. Mater.* **21**, 2896-2900 (2011).

3. Hwang, S.-H., Wang, P., Moorefield, C. N., Godínez, L. A., Manríquez, J., Bustosc, E. & Newkome, G. R. Design, self-assembly, and photophysical properties of pentameric metallomacrocycles: [M_5(N-hexyl[1,2-bis(2,2′:6′,2″-terpyridin-4-yl)]carbazole)$_5$][M = Fe(II), Ru(II), and Zn(II)]. *Chem. Commun.* **37**, 4672-4674 (2005).

4. Maity, D., Bhaumik, C., Mondal, D. & Baitalik, S. Ru(II) and Os(II) complexes based on terpyridyl-imidazole ligand rigidly linked to pyrene: synthesis, structure, photophysics, electrochemistry, and anion-sensing studies. *Inorg. Chem.* **52**, 13941-13955 (2013).

5. Shunmugam, R., Gabriel, G. J., Aamer, K. A. & Tew, G. N. Metal-ligand-containing polymers: terpyridine as the supramolecular unit. *Macromol. Rapid Commun.* **31**, 784-793 (2010).

6. Nie, H.-J., Yao, J. & Zhong, Y.-W. Synthesis of vinyl-substituted polypyridyl ligands through suzuki miyaura cross-coupling of potassium vinyltrifluoroborate with bromopolypyridines. *J. Org. Chem.* **76**, 4771-4775 (2011).

7. Zakeeruddin, S. M., Nazeeruddin, M. K., Pechy, P., Rotzinger, F. P., Humphry-Baker, R., Kalyanasundaram, K. & Grätzel, M. Molecular engineering of photosensitizers for nanocrystalline solar cells: synthesis and characterization of Ru dyes based on phosphonated terpyridines. *Inorg. Chem.* **36**, 5937-5946 (1997).

8. Troian-Gautier, L., DiMarco, B. N., Sampaio, R. N., Marquard, S. L. & Meyer, G. J.

Evidence that $\Delta S^{\ddagger}$ controls interfacial electron transfer dynamics from anatase TiO_2 to molecular acceptors. *J. Am. Chem. Soc.* **140**, 3019-3029 (2018).
